# Should I stay or should I go? Causes and consequences of intraspecific variation in site fidelity

**DOI:** 10.1186/s40462-025-00606-w

**Published:** 2025-11-06

**Authors:** Katey S. Huggler, Rachel A. Smiley, Brittany L. Wagler, Alyson B. Courtemanch, Zach Gregory, Kevin L. Monteith, Lisa A. Shipley, Cheyenne Stewart, Paul Wik, E. Frances Cassirer, Ryan A. Long

**Affiliations:** 1 Department of Fish and Wildlife Sciences, University of Idaho, 875 Perimeter Drive, MS 1136, Moscow, ID 83844 USA; 2 Haub School of the Environment and Natural Resources, University of Wyoming, 804 E. Fremont Street, Laramie, WY 82071 USA; 3 Wyoming Game and Fish Department, 420 N Cache Street, Jackson, WY 83001 USA; 4 Wyoming Game and Fish Department, 260 Buena Vista Drive, Lander, WY 82520 USA; 5 Haub School of the Environment and Natural Resources, Wyoming Cooperative Fish and Wildlife Research Unit, University of Wyoming, 804 E. Fremont Street, Laramie, WY 82071 USA; 6 School of the Environment, Washington State University, 1229 Webster Hall, Pullman, WA 99164 USA; 7 Washington Department of Fish and Wildlife, 1049 Port Way, Clarkston, WA 99403 USA; 8 Idaho Department of Fish and Game, 3316 16th Street, Lewiston, ID 83501 USA; 9 Present Address: Cooperative Wildlife Research Unit, University of Montana, 205 Natural Sciences Building, Missoula, MT USA; 10 Present Address: Asotin County Fire District #1, 2377 Appleside Boulevard, Clarkston, WA 99403 USA

**Keywords:** Bighorn sheep, Constancy, Foodscape, Nutritional condition, Philopatry, Predictability, Suitable biomass, Survival

## Abstract

**Background:**

Site fidelity, the tendency to return to previously visited locations, is common across a wide range of taxa and ecosystems. Site fidelity can benefit animals by improving foraging efficiency, reducing movement costs, and increasing reproductive success. Nevertheless, considerable variation exists within and among species in the nature and magnitude of site fidelity, and the mechanisms underpinning this variation are poorly understood. One hypothesis for explaining variation in site fidelity suggests that in predictable resource landscapes, fidelity should be conditional on prior reproductive success (win-stay, lose-switch). Alternatively, animals occupying less predictable resource landscapes should make greater use of cues from their current environment and site fidelity should scale inversely with the magnitude of environmental heterogeneity.

**Methods:**

We investigated the causes (e.g., prior reproductive success, foodscape heterogeneity) and consequences (e.g., nutritional condition, neonate survival) of intraspecific variation in site fidelity during spring and summer among three bighorn sheep (*Ovis canadensis*) populations occupying a low-elevation grassland and two alpine ecosystems. We used distance-based metrics to quantify site fidelity at inter-annual, inter-month and inter-week scales to better understand the behavioral strategies employed by bighorn sheep to mitigate environmental variation and optimize foraging opportunities.

**Results:**

Site fidelity varied considerably among study areas and across temporal scales. Prior reproductive success was not an important predictor of site fidelity by bighorn sheep, and instead, site fidelity appeared to be influenced by quality and predictability of forage resources within individual home ranges. Despite consistency of this effect, however, we found little evidence that site fidelity improved nutritional condition of female sheep or neonate survival to 120 days.

**Conclusions:**

Our results generally support the notion that environmental conditions shaped the strength of site fidelity across temporal scales. Yet, the benefits of site fidelity were limited, at least based on the performance metrics we measured. Continuing to parse the complex mechanisms underpinning variation in site fidelity will shed important light on the capacity of animals to adjust to an unpredictable and changing environment.

**Supplementary Information:**

The online version contains supplementary material available at 10.1186/s40462-025-00606-w.

## Background

Animal movements can influence ecological patterns and processes across scales and levels of organization, from ecosystem-wide rates of nutrient cycling [1–4] to the nature and magnitude of competitive interactions within and among species [5–7]. Site fidelity, the tendency to re-visit familiar areas [8], is a common movement strategy that is found across a wide range of taxa [9, 10]. The fitness benefits of site fidelity generally derive from an animal’s knowledge of its physical and social environment [8, 11, 12]. Familiarity with previously used sites can confer numerous advantages, including efficient resource acquisition [13], improved defense against competitors [8, 14], and knowledge of predation risk [15]. Despite the potential benefits of site fidelity, however, unpredictable patterns of resource distribution or risk can cause this strategy to be maladaptive in heterogeneous landscapes [12, 16–18]. This apparent tradeoff has generated increased interest in understanding the degree to which site fidelity is a plastic behavioral trait. For example, Lafontaine et al. [12] showed that female caribou (*Rangifer tarandus*) were extremely faithful to summer ranges because site familiarity reduced predation on calves by opportunistic predators, but were less faithful to winter ranges, which helped to reduce predation by wolves (*Canis lupus*). Such empirical examples are limited, however, and thus much remains to be learned about the causes and consequences of plasticity in site fidelity.

The strength of site fidelity also is scale-dependent, and fidelity can be measured across multiple spatial (e.g., nest sites, home ranges, or territories; [16, 19]) and temporal (e.g., days, seasons, or years) scales. Individuals may exhibit less consistent patterns of behavior at finer temporal scales (e.g., weekly or monthly) in response to variation in resources or predation risk [12, 19]. For example, home-range fidelity of female elk (*Cervus canadensis*) in central Canada was positively related to forage availability across temporal scales, but at fine scales (i.e., daily to biweekly) elk were less faithful to areas with high forage abundance when predation risk was high [19]. Such results highlight the importance of considering scale when measuring site fidelity.

Home ranges—the area traversed by an animal for food gathering, mating, and provisioning of young [20]—are an emergent property of animal movements, and understanding the causes and consequences of variation in the size, distribution, and quality of home ranges is critical for linking movements to population performance. Optimal foraging theory predicts that animals should occupy the minimum area needed to gather adequate resources for supporting survival and reproduction while reducing time and energy allocated to resource defense and foraging [21–23]. Accordingly, the spatiotemporal distribution of forage resources should at least partly determine optimal strategies for obtaining those resources [24, 25], and ultimately should drive variation in home-range size [26, 27]. Specifically, animals that occupy landscapes that are relatively homogeneous in space or time should have smaller home ranges than animals that live in heterogeneous or less predictable landscapes; heterogeneous resource distributions require animals to move more among patches, leading to larger home ranges [26–29]. Empirical data on relationships between resource distribution and home-range size have largely supported this prediction [28, 30, 31]. In contrast, the influence of resource distribution on animals’ propensity to return to seasonal home ranges (i.e., site fidelity) remains largely unexplored [8, 16, 32].

The decision to be faithful to a home range has most often been linked to prior reproductive success [16, 33, 34]. Under the win-stay, lose-switch (WSLS) rule, individuals should utilize ‘private’ knowledge (i.e., knowledge gained through individual experience) and remain faithful to a home range if they were previously successful with respect to some metric that impacts fitness (e.g., offspring survival), whereas they should shift their home range if they were unsuccessful [16, 34]. The WSLS rule, however, does not account for public knowledge (i.e., knowledge shared by all individuals) about factors such as variation in resource availability [16, 33, 35]. Areas with high spatiotemporal variation (i.e., low predictability) in resources provide little basis for predicting the future, and therefore decisions about home range use should be independent of experience (i.e., site fidelity should be low regardless of prior reproductive success; [16, 35]). Conversely, when resources are more predictable across space and time, animals should exhibit higher fidelity to profitable patches, especially when movement costs are high [16].

Spatial and temporal variation in forage resources can influence patterns of site fidelity in distinct and scale-dependent ways. When spatial variation in forage quantity and/or quality is high, animals should use the same locations more consistently across time [36]—resulting in strong site fidelity. In such scenarios, spatial predictability should play a key role in shaping movement and space-use decisions, especially at fine temporal scales (e.g., weeks or months). Conversely, when forage quality and/or availability is spatially uniform but temporally variable—for example, when the same areas fluctuate from productive to unproductive across years owing to unpredictable rainfall or disturbance—animals may show weaker fidelity, because returning to previously used sites carries a greater risk of encountering unfavorable foraging conditions. In these scenarios, temporal predictability should be the key driver of fidelity at broader temporal scales (e.g., years). Thus, spatial variation in forage should have a stronger effect on site fidelity at fine temporal scales, whereas temporal variation should have a greater influence at broad temporal scales. Disentangling the relative contributions of spatial and temporal predictability is key to understanding how site fidelity emerges and varies across ecological contexts.

Bighorn sheep (*Ovis canadensis*) are an excellent species for testing hypotheses about plasticity in site fidelity because they inhabit a wide variety of ecosystems, from deserts to high-elevation alpine environments. Such diversity provides an opportunity for evaluating whether and to what degree resource heterogeneity influences the causes and consequences of site fidelity. Here, we investigate intraspecific variation in site fidelity among three bighorn sheep populations inhabiting different environments. One population (Asotin Creek, Washington, USA) occupied a relatively low-elevation grassland that was characterized by long, hot and dry summers and where spring precipitation was an important determinant of inter-annual variation in forage availability and quality. The other two populations (Whiskey Mountain and Jackson, Wyoming, USA) were composed of seasonal, elevational migrants that occupied alpine environments that were strongly seasonal with cool, reliably wet summers and long cold winters, and that were more topographically complex. We integrate key concepts from optimal foraging theory, nutritional ecology, and movement ecology to evaluate relationships among the foodscape, site fidelity and metrics of performance (i.e., nutritional condition and neonate survival) in all three populations. We tested two overarching hypotheses: first that intraspecific variation in site fidelity is influenced by predictability of forage resources across space and time, and second that site fidelity influences nutritional condition and reproductive success. We tested the following predictions relating to summer (April – September) site fidelity of female bighorn sheep derived from those hypotheses:

1. Forage resources in the grassland environment will be more temporally unpredictable than in the alpine environment because forage in the grassland is dependent on spring precipitation, which varies substantially from year to year. In contrast, we expect forage in the grassland to be more spatially homogeneous, because the relatively simple topography of the grassland promotes a more uniform distribution of vegetation than the highly variable terrain of alpine environments.
2. Bighorn sheep in the grassland environment will exhibit lower site fidelity across temporal scales because forage will be less predictable, which should necessitate more extensive movements to locate resources. In contrast, bighorn sheep in the alpine environment will exhibit higher site fidelity because individuals are migratory and forage availability is more temporally stable owing to the predictable seasonality of the alpine.
3. Site fidelity of bighorn sheep in the less temporally predictable grassland environment will be strongly and inversely related to heterogeneity in the foodscape. Conversely, in the more temporally predictable alpine environments, site fidelity will primarily be determined by (and positively related to) prior reproductive success (i.e., WSLS rule).
4. Females that show higher site fidelity during summer in the more (temporally) predictable alpine environments will enter winter in better nutritional condition than females that are less faithful, whereas site fidelity will not influence nutritional condition in the less (temporally) predictable grassland environment.
5. Neonates born to females that are faithful to summer home ranges in the more temporally predictable alpine environments will have a higher probability of surviving their first 120 days of life, whereas site fidelity will not influence neonate survival in the less (temporally) predictable grassland environment.

## Methods

### Study area

**Fig. 1:**
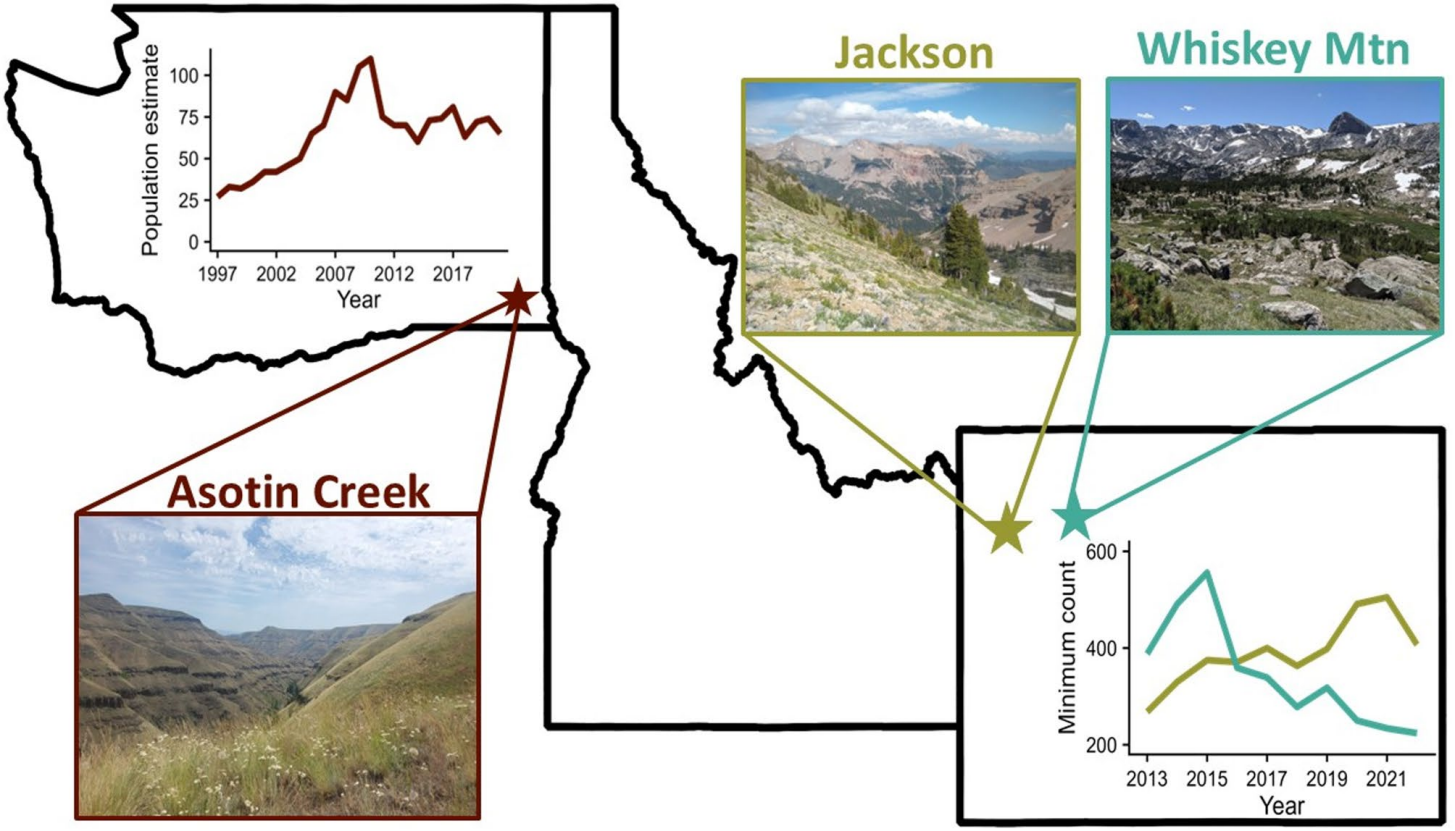
Study area locations (stars) near Asotin Creek, Washington, USA (red), Jackson, Wyoming, USA (gold), and Whiskey Mountain, Wyoming, USA (teal). Figures inside state silhouettes show population trends from 1997–2022 in Asotin Creek and from 2013–2022 in Jackson and Whiskey Mountain

Our study included three summer ranges of bighorn sheep: Hells Canyon (46°17’N, 117°16’W) near Asotin, Washington, USA (hereafter, Asotin Creek), Jackson (43°31’N, 110°29’W) near Jackson, Wyoming, USA, and Whiskey Mountain (43°25’N, 109°37’W) near Dubois, Wyoming, USA (Fig. 1). The Asotin Creek study area was a low-elevation grassland environment that encompassed a 240-km^2^ herd range and was characterized by steep, rugged Columbia River basalts and communities of perennial bunchgrasses (*Pseudoroegnaria spicata* and *Festuca idahohensis*) and annual grasses (*Bromus* spp.), patchworks of riparian shrubs (*Philadelphus lewisii* and *Crataegus douglasii*), and mixed conifers (*Pseudotsuga menziesii* and *Pinus ponderosa*) on north-facing slopes [37]. Elevations in Asotin Creek ranged from 428 to 1170 m. Asotin Creek was an arid landscape with a dry climate (median April–September precipitation 1991–2020, 39.8 cm), and winters were generally mild (mean January temperature = 5.6 °C, snow depth = 20.3 cm; SNOTEL, Sourdough Gulch Station). During summer 2021, Asotin Creek experienced a drought (May 2021 precipitation = 23% of median; SNOTEL, Sourdough Gulch Station) and a large wildfire (the Lick Creek fire) that burned 95% of the bighorn summer range in July, leaving little vegetation for the remainder of the summer. In contrast, during spring 2022, Asotin Creek experienced record-breaking precipitation (May 2022 precipitation = 178% of median; SNOTEL, Sourdough Gulch Station).

The summer ranges of both the Jackson and Whiskey Mountain herds were high-elevation alpine ecosystems that encompassed approximately 300 km^2^ and 450 km^2^, respectively [38, 39]. Both study areas were characterized by rugged, rocky peaks at ~ 3500–4000 m that transitioned to winter range at ~ 2200 m [38, 39]. High elevations throughout the Jackson and Whiskey Mountain summer ranges were typified by alpine meadows and talus scree. Mid to low elevations contained interspersed patches of mixed conifers (e.g., *Pinus flexilis* and *Abies* spp.), mountain shrubs (e.g., *Ericameria suffruticosa* and *Dasiphora fruiticosa*), and grasslands (e.g., *Poa secunda*) [38, 39]. Low elevations (winter range for bighorn sheep) were composed of mixed grasses and sagebrush. The Jackson and Whiskey Mountain study areas were characterized by short, cool summers (mean April–September precipitation ranging from 43.2 to 48.3 cm, SNOTEL; Cold Springs and Gros Ventre Summit Stations) and long, often severe winters with mean January temperatures ranging from − 16 to -7 °C and mean snow depth ranging from 40.6 to 66.4 cm (SNOTEL; Cold Springs and Gros Ventre Summit Stations). Bighorn sheep residing in both these study areas were seasonal, elevational migrants [39].

In addition to bighorn sheep, the ungulate community in Asotin Creek included mule deer (*Odocoileus hemionus*), white-tailed deer (*Odocoileus virginianus*), elk, and domestic cattle (*Bos taurus*) that grazed primarily on private land and periodically on public land. The Jackson and Whiskey Mountain summer ranges supported populations of elk and Shiras moose (*Alces alces shirasi*); mule deer occur throughout the Jackson range, but only at low elevations in Whiskey Mountain. Predators in all study areas included mountain lions (*Puma concolor*), bobcats (*Lynx rufus*), coyotes (*Canis latrans*), black bears (*Ursus americanus*), gray wolves (*Canis lupus)*, and golden eagles (*Aquila chrysaetos*). Grizzly bears (*Ursus arctos horribilis*) were present in Jackson and at low densities at Whiskey Mountain.

The reintroduced Asotin Creek population is part of the Hells Canyon metapopulation that spans portions of Washington, Idaho and Oregon, USA. The Asotin Creek herd was initially established by translocations of 29 bighorn sheep from 1973 to 1997; 19 sheep from Washington, USA and 10 sheep from British Columbia, Canada [40]. At its peak in 2011, the Asotin Creek population contained approximately 105 sheep. However, following an all-age die-off from pneumonia in 2012, the population declined to ~ 60 individuals by 2014, and during this study the estimated population ranged from 65 to 75 sheep [41] (Fig. 1).

The Jackson and Whiskey Mountain bighorn sheep populations are native core herds in Wyoming (i.e., they have never been extirpated or augmented). All-age die-offs attributed to pneumonia occurred in Jackson in the early 2000s, causing a loss of >50% of the herd. The Jackson herd has since recovered to its pre-outbreak abundance of ~ 300–500 individuals and has maintained high neonate recruitment (Fig. 1) [42–45]. In contrast to Jackson, the Whiskey Mountain population was once the largest bighorn sheep herd in Wyoming and served as the source population for many translocation efforts. However, following massive die-offs from pneumonia in the late 1990s, followed by decades of low recruitment, the population has declined to roughly 20% of its historic abundance at approximately 200 individuals (Fig. 1) [46–49].

### Capture, handling, and monitoring

We captured adult (i.e., ≥ 1.5 years old) female bighorn sheep in Asotin Creek during October–November, 2019–2022 (Table 1) by darting from a vehicle (0.75 ± 0.15 mg/kg butorphanol, 0.25 ± 0.05 mg/kg azaperone, and 0.30 ± 0.06 mg/kg medetomidine; BAM; [50]). We captured female bighorn sheep in Wyoming during March and December 2019–2022 using a hand-held net gun fired from a helicopter [51, 52] and transported them to a central processing location for data collection. In all study areas, we hobbled (except for chemically immobilized sheep) and blindfolded sheep to minimize stress.

**Table 1:** Sample sizes of Bighorn sheep (adult females and neonates) monitored at Asotin Creek, Washington, USA, and Jackson and whiskey Mountain, Wyoming, USA, 2019–2022. Adult sample sizes include females that were monitored during multiple years

	Asotin Creek		Jackson		Whiskey Mountain	
Year	Adults	Neonates	Adults	Neonates	Adults	Neonates
2019	16	0	16	4	27	13
2020	19	5	17	9	20	10
2021	19	15	16	7	21	12
2022	15	16	24	15	13	3
Total	69	36	73	35	81	38

During autumn captures in all study areas, we quantified nutritional condition of bighorn sheep using a combination of ultrasonography and palpation scoring [53]. We used ultrasonography (Ibex Pro, EI Medical Imaging, Loveland, Colorado USA) to measure thickness of subcutaneous rump fat and of the bicep and loin muscles to the nearest 1 mm [53]. Additionally, we estimated a condition score via palpation of the sacrosciatic ligament and caudal vertebrae [53]. Finally, we combined measurements of fat thickness and body condition to estimate percent ingesta-free body fat (IFBFat) using equations developed by Stephenson et al. [53] for bighorn sheep. We determined reproductive status of females using one of two methods: (1) females were designated as lactating or recently weaned at capture if they had a surviving neonate (determined from neonate capture and monitoring, details below), or (2) when we did not have data on recruitment status from radiomarked neonates, we determined lactation status by palpating the udder and attempting to express milk [39, 54]. Bighorn sheep in Wyoming were re-captured during spring (mid-March), at which time all data described above were collected again, pregnancy was assessed via ultrasonography, and pregnant females were fit with a vaginal implant transmitter (VIT; Vectronic Aerospace, Berlin, Germany) to aid in subsequent capture of neonates (details below).

We fit bighorn sheep with an Iridium GPS collar (Vectronic Aerospace, Berlin, Germany) programmed to collect locations at 1-h intervals. We truncated GPS data based on differences in the onset of spring and associated timing of parturition and plant phenology between the grassland and alpine study sites: 1 April–1 September in Asotin Creek, and 1 June–1 September in Jackson and Whiskey Mountain. We removed locations with unrealistic step lengths (i.e., greater than the 95th percentile) to help ensure that erroneous locations were not included in the GPS dataset [55]. We partitioned GPS data into temporal bins at three different resolutions: inter-annual (limited to the date ranges described above for each study area), inter-month (30-day intervals within each year), and inter-week (14-day intervals within each year). Furthermore, only females that survived at least 50% of the year, month or 14-day interval (depending on the scale of analysis) were included in analyses.

To quantify the effects of breeding success on site fidelity and the effects of site fidelity on neonate survival, we captured and monitored neonates born to GPS-collared females in Asotin Creek, Jackson, and Whiskey Mountain from 2019 to 2022 (Table 1). In Wyoming, we used VITs to identify the timing and location of parturition. VITs were equipped with a UHF communication system that sent notification of VIT expulsion via the female’s GPS collar, which allowed us to capture neonates quickly after birth. In Asotin Creek, where we did not use VITs, we identified parturient females by observing each marked female daily with high-powered optics. We captured neonates within 48 h of birth during peak parturition (May–June), determined sex, and measured body mass, body length, chest girth, and metatarsus length. We fit neonates with either an expandable VHF collar (Asotin Creek; Advanced Telemetry Systems, Isanti, MN, USA) or a GPS collar (Wyoming; Vectronic Aerospace, Berlin, Germany) programmed to send a mortality signal after 4 h of inactivity. We monitored survival of neonates daily via radio telemetry in Asotin Creek and via remote monitoring of GPS collar notifications in Wyoming. We investigated mortalities within 24 h whenever possible.

### Modeling the foodscape

During spring and summer, 2021–2022, we conducted intensive vegetation sampling at Asotin Creek, Jackson, and Whiskey Mountain to quantify spatiotemporal variation in energy and protein available to bighorn sheep. We used clip-and-weigh methods and a double-sampling approach to quantify forage biomass at transects distributed throughout each study area, and simple linear regression to predict biomass at unclipped locations as a function of percent cover and other covariates (Additional file 1: Appendix 1). We collected samples of plant species and parts at the same transect locations and kept them on ice until they could be frozen. We then freeze-dried forbs and shrub samples and air-dried graminoid samples, ground samples, and analyzed for neutral detergent fiber (NDF, %), acid detergent lignin (ADL, %), acid insoluble ash (AIA, %) using sequential fiber analysis, sodium sulfite (for non-graminoids) and the Ankom filter bag method. Additionally, we analyzed a subset of samples for gross energy (GE; kJ g^− 1^) using bomb calorimetry, nitrogen (%) from the Dumas combustion method, and protein-precipitating capacity of condensed tannins (mg Bovine Serum Albumin [BSA] precipitate/mg forage) using the Martin and Martin [56] assay. Nutritional analyses were conducted at Dairy One Forage Laboratory, Ithaca, NY, USA, Idaho Department of Fish and Game Wildlife Health Laboratory Boise, ID, USA, and Washington State University Wildlife Habitat Laboratory, Pullman, WA, USA.

We estimated dry matter digestibility (DMD, %) by entering estimates of NDF, ADL, AIA, and BSA into summative equations developed by Robbins et al. [57] for deer (*Odocoileus* spp.) and elk. We then calculated the digestible energy (DE) content (kJ g-1) of each forage sample as the product of GE and DMD. We calculated digestible protein (DP) content (g protein/100 g forage) of each forage sample by entering estimates of crude protein (CP, 6.25 × nitrogen content) and BSA into the corresponding equation from Robbins et al. [57]. We integrated data on forage biomass and quality (i.e., DE and DP) to estimate suitable forage biomass, defined as the minimum biomass of forage that together meets nutritional requirements for supporting a specified level of performance (e.g., lactation), using the Forage Resource Evaluation System for Habitat (FRESH) model [58]. Finally, we used generalized additive models (GAMs; [59]) to evaluate relationships between suitable biomass and remotely sensed covariates (e.g., NDVI, climate, topography) and to predict spatiotemporal variation in the foodscape (i.e., suitable forage biomass) within home ranges of bighorn sheep at Asotin Creek, Jackson, and Whiskey Mountain during spring and summer, 2019–2022 [60]. We used the top model for each study area to generate 250-m resolution rasters of daily suitable biomass at each site. A detailed description of our approach to modeling the foodscape is provided in Additional file 1: Appendix 1.

### Estimating home ranges

We estimated home ranges at inter-annual, inter-month (30 days), and inter-week (14 days) scales using 99% utilization distributions derived from dynamic Brownian bridge movement models (dBBMM; [61]). We chose dBBMMs because they allowed us to probabilistically characterize the environmental (both spatial and temporal) predictability an individual likely experienced within the area they traversed during specific temporal windows. While we acknowledge that dBBMMs may be described more accurately as occurrence distributions than traditional home ranges [62], for simplicity and consistency we refer to dBBMM-derived estimates as ‘home ranges’” throughout. We calculated seasonal dBBMMs for each animal and year. When fitting a dBBMM to each bighorn sheep movement trajectory, we specified an error of 20 m. Also, as recommended by Kranstauber et al. [61], we specified a small moving window size of 7 locations (7 h) and a margin of 3 locations (3 h) to ensure that we captured short-term changes in movement variation. We estimated home range size at each temporal scale as the area (km^2^) under the 99% contour. We used the ‘move’ package in Program R to estimate all dBBMM home ranges [61, 63].

### Environmental predictability and quality

As a measure of environmental predictability, we quantified spatial and temporal ‘constancy’ of suitable forage biomass within sheep home ranges at inter-annual, inter-month, and inter-week scales. At each temporal scale, we clipped model-predicted suitable biomass layers to individual home ranges. Metrics of spatial constancy quantified heterogeneity of suitable biomass within an individual’s home range during a given time period, whereas temporal constancy quantified the stability of suitable biomass within a home range through time [9]. We scaled all suitable biomass layers across all study areas so that values varied between 0 and 1, and metrics of quality and predictability could be compared across study areas. We calculated Colwell’s [64] constancy measure, C, based on Shannon’s [65] entropy, H, as follows:

1 $$\:C=1-H/log\:log\:\left(n\right)$$

and:

2 $$\:H=\:\sum\limits_{i=1}^{n}{P}_{i}\times\:log\left({P}_{i}\right)$$

**Fig. 2:**
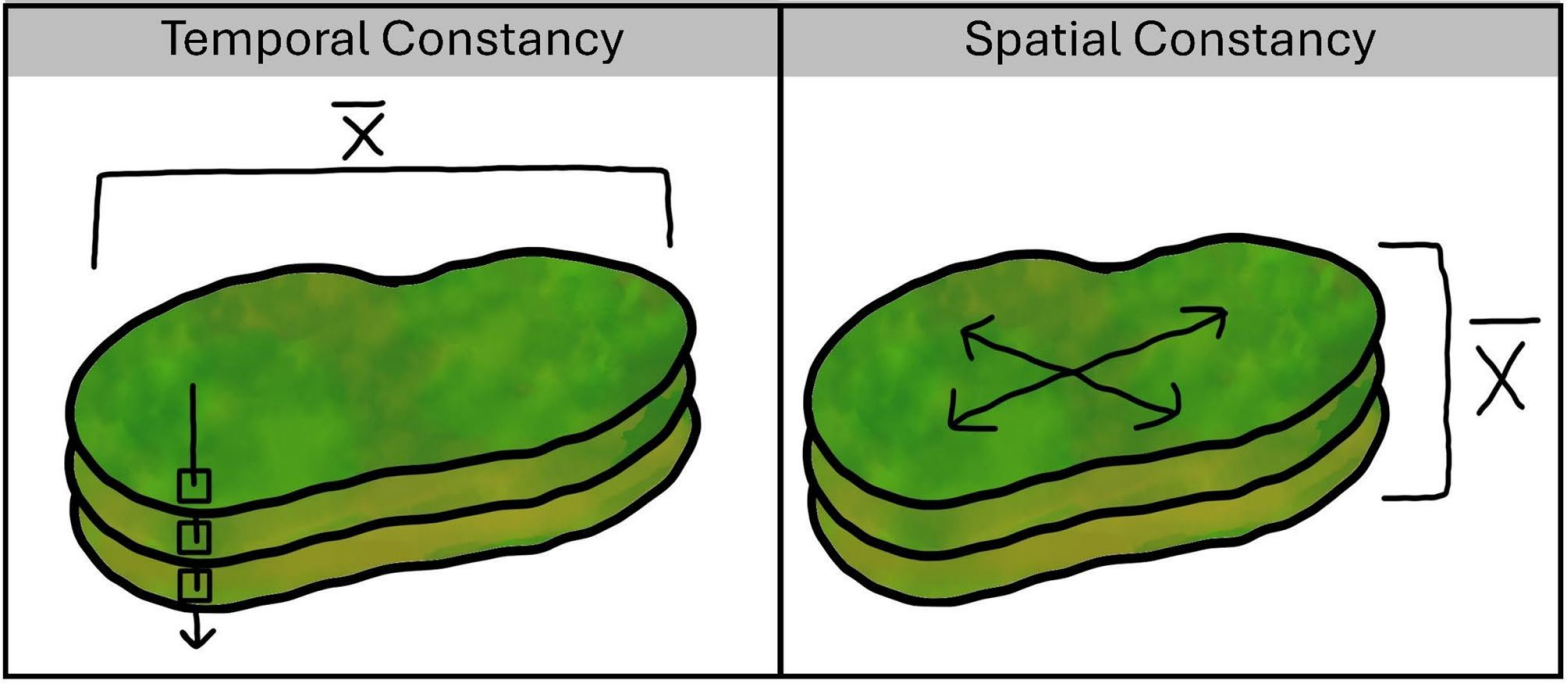
Conceptual representation of temporal (left) and spatial (right) constancy calculations within a hypothetical home range. We calculated temporal constancy across days within a year, month, or week on a per-pixel basis, and then averaged ($\bar{x}$) constancy values across pixels within the home range. We calculated spatial constancy on a per-home range basis and then averaged home-range-level values across days within each season, month, or week

Here, *P* is the proportion of suitable biomass values that fall within interval *i* across *n* intervals. We calculated *temporal constancy* on a per-pixel basis via the following steps. First, we created *n* = 100 intervals that were evenly spaced between the minimum and maximum values of suitable biomass (0 and 1 in this case). We then calculated the proportion of daily suitable biomass estimates, *P*, for each pixel, *x*, that fell within each interval, *i*. We fed those results into the equations above for calculating *H* and *C*, and the final product was a single layer that contained estimates of temporal constancy, *C*, for each pixel in each sheep home range (Fig. 2). We averaged values of *C* across pixels within each home range to estimate temporal constancy for each animal at each temporal scale. Similarly, we calculated *spatial constancy* on a per-home-range basis. We calculated the proportion of pixels in each home range that fell within each interval, *i*, and input those proportions into the equations above for calculating *H* and *C*. This resulted in an estimate of spatial constancy for each home range and day within the time period of interest. We then calculated the average spatial constancy across days at each temporal scale (Fig. 2). Both temporal and spatial constancy values ranged from 0 to 1, which corresponded to high and low levels of environmental variation, respectively. Separate from analyses of constancy, we also calculated mean suitable biomass (scaled) within each individual home range and temporal scale of analysis as an indicator of home-range quality.

### Quantifying site fidelity across temporal scales

We quantified inter-annual, inter-month, and inter-week site fidelity using distance-based metrics described by Morrison et al. [9]. In this case, distance is inversely related to site fidelity (i.e., greater distance corresponds to lower site fidelity). We chose a distance-based metric over indices of home range overlap because it allowed us to quantify spatial proximity on an absolute scale and in units (meters) with direct relevance to management and conservation [9]. At the inter-annual scale, we defined ‘sites’ as the set of GPS locations visited during year *t*. We quantified ‘site fidelity’ as the mean minimum Euclidean distance between each location in year *t* and the subset of locations in year *t* – 1 that fell within a specified time window (in days) of the location in year *t* (i.e., locations collected around the same time during the previous year; [9, 66]). To optimize window size, we conducted a sensitivity analysis across a range of window sizes from 5 to 100 days in increments of 5 days, and plotted window size against the mean and coefficient of variation of inter-annual distance for each window. We selected the most appropriate window size for each study area by visually inspecting the plots and identifying the window size at which an asymptote was reached for both the mean and coefficient of variation in inter-annual distance ([9]; Additional file 2: Appendix 2). We quantified inter-month site fidelity as the mean minimum distance between all spatial locations (*x*,* y*) for individual *i* in month *m* and all spatial locations for that same individual in month *m* – 1. Similarly, we quantified inter-week site fidelity as the mean minimum distance between all spatial locations (*x*,* y*) for individual *i* in week *w* and all spatial locations for that individual in week *w* – 1.

### Defining recruitment status

We used neonate survival data to define recruitment status across temporal scales and to evaluate the effects of reproductive success on site fidelity. Specifically, we defined recruitment status in the previous timestep (i.e., year, month, or week) as the predictor of site fidelity. At the inter-annual scale, we considered females whose offspring survived to 120 days of age in year *t-1* to have successfully recruited. At the inter-month scale, females whose offspring survived ≥ 15 days during the previous 30-day period were considered to have a neonate at heel during that period. Finally, at the inter-week scale, females whose offspring were known to be alive for at least 7 days during the previous 14-day period were considered to have a neonate at heel during that period.

### Modelling approach

We used linear mixed-effects models to assess how the environment (i.e., public knowledge) and experience (i.e., private knowledge) influenced inter-annual, inter-month, and inter-week site fidelity. Candidate model sets reflected our hypotheses and included covariates related to (1) resource heterogeneity, (2) previous success, or (3) resource heterogeneity and previous success combined. Within each model set, we fit a series of sub-models that included additive and interactive effects of public and/or private knowledge. In the resource heterogeneity model set, we tested four competing sub-models that represented the effects of (1) predictability (metrics of temporal and spatial constancy), (2) quality (mean suitable biomass), (3) predictability + quality, and (4) predictability × quality, on site fidelity. In the previous success model set, we fit a single model that included recruitment status from the previous year, month, or week (depending on the scale of analysis). Our model set representing the combined effects of resource heterogeneity and previous success included six sub-models: (1) predictability + recruitment, (2) quality + recruitment, (3) predictability + quality + recruitment, (4) predictability × recruitment, (5) quality × recruitment, and (6) predictability × recruitment + quality × recruitment. We log-transformed the dependent variable (i.e., fidelity distance [meters]) to ensure normality and to adhere to model assumptions. We deemed an effect to be significant if the model-averaged 95% confidence interval did not overlap zero [67–71].

We expected there to be a strong, negative effect of home range size on site fidelity, and we controlled for this by including a fixed effect for home range size (km^2^) in all models. We standardized all covariates to facilitate direct comparison of effect sizes [72]. Prior to model fitting we screened variables for collinearity (|*r* | >0.6), and when covariates within a sub-model were collinear, we split the sub-model into multiple components. For example, if spatial and temporal constancy were collinear in a sub-model that included effects of predictability, we fit separate models for each constancy metric. We included a random intercept for animal ID at the inter-annual scale and for animal ID-year at the inter-month and inter-week scales [73]. We based inferences on model-averaged coefficients from the 95% confidence set of models [74] estimated using the ‘AICcmodavg’ package in Program R [74]. With only a single exception (a weak predictability × quality interaction at Whiskey Mountain), none of the interaction terms were significant in any model sets. Accordingly, we elected to interpret model-averaged coefficients from the additive model set only and excluded interactions. We present model-averaged coefficients from all additive models in Tables 2, 3 and 4.

### Linking site fidelity to nutritional condition

We used linear mixed-effects models to relate site fidelity to autumn nutritional condition of bighorn sheep at Asotin Creek (grassland), and Jackson and Whiskey Mountain (alpine). Furthermore, at Jackson and Whiskey Mountain, where we had paired estimates of body fat in spring and autumn, we fit additional linear mixed-effects models that related site fidelity to fat accretion (absolute change in body fat) between spring and autumn. Because regulation of somatic reserves is state-dependent (i.e., a function of body fat at the beginning of the season; [38, 75, 76]) we accounted for initial state by including estimates of spring body fat in our models of fat accretion in Wyoming. We fit separate models for each temporal scale of analysis, and considered the effects of site fidelity, recruitment status, and spring fat (fat accretion models only) and the 2-way interaction between site fidelity and recruitment status, because we expected that recruitment status would modulate the relationship between metrics of energy storage and site fidelity. We could not estimate the interaction between recruitment status and site fidelity at Whiskey Mountain because too few females at that site successfully recruited a neonate. At the inter-annual scale, we used the single estimate of mean inter-annual distance (log-meters) as the metric of site fidelity. At the inter-month and inter-week scales, we calculated the mean minimum inter-month or inter-week distance within a season as an indicator of average site fidelity. We included a random intercept for animal ID in all models to account for repeated measures of autumn body fat or fat accretion on individuals across years. All mixed-effects models were fit using the ‘lme4’ package in Program R version 4.3.0 [77].

### Linking site fidelity to juvenile survival

Juvenile survival to 120 days in Asotin Creek was high (*n* = 6 mortalities in 3 years), and thus we did not have sufficient sample size to model neonate survival in that study area. Consequently, we used an unpaired two-sample Mann-Whitney test to compare inter-annual site fidelity of ewes with neonates that survived and ewes with neonates that did not survive. For our alpine study areas in Wyoming, we used a continuous time-to-event approach to model the effects of intrinsic and extrinsic factors on survival of bighorn neonates to 120 days, using birth as the origin [78, 79]. We right-censored neonates with failed collars (*n* = 2 and *n* = 1 in Whiskey Mountain and Jackson, respectively) and neonates that survived beyond the 120-day window (*n* = 5 and *n* = 16 in Whiskey Mountain and Jackson, respectively). We used the Andersen-Gill formulation of the Cox proportional hazards model [80, 81] to evaluate factors influencing risk of mortality. The model takes the form:

3 $$\:h\left({x}_{i}\right)=\:{h}_{0}\left(t\right){e}^{{x}_{i\beta\:}}$$

The model makes no distributional assumptions for the baseline hazard $\:{h}_{0}\left(t\right)$, and is semi-parametric [79, 82]. Exponentiated coefficients *e*^*β*^ are interpreted as hazard ratios (HR) wherein HR < 1 indicates the hazard of mortality decreases as the covariate increases and HR >1 indicates the hazard of mortality increases with increasing covariate values.

Because we were specifically interested in evaluating the influence of site fidelity by the maternal female on neonate survival, we limited candidate covariates in our survival models to metrics of mean fidelity. To account for repeated measures on individuals, we included a cluster for animal ID [78]. We used the survival package in Program R version 4.3.0 [83] for Cox proportional hazards modeling, including assessment of the proportional hazards assumption. We conducted all lamb survival analyses at the inter-annual scale because metrics of site fidelity were highly correlated across temporal scales, and because juvenile survival over the first 120 days is likely a product of maternal behavior over the entire season.

## Results

### Site fidelity – general patterns

**Fig. 3:**
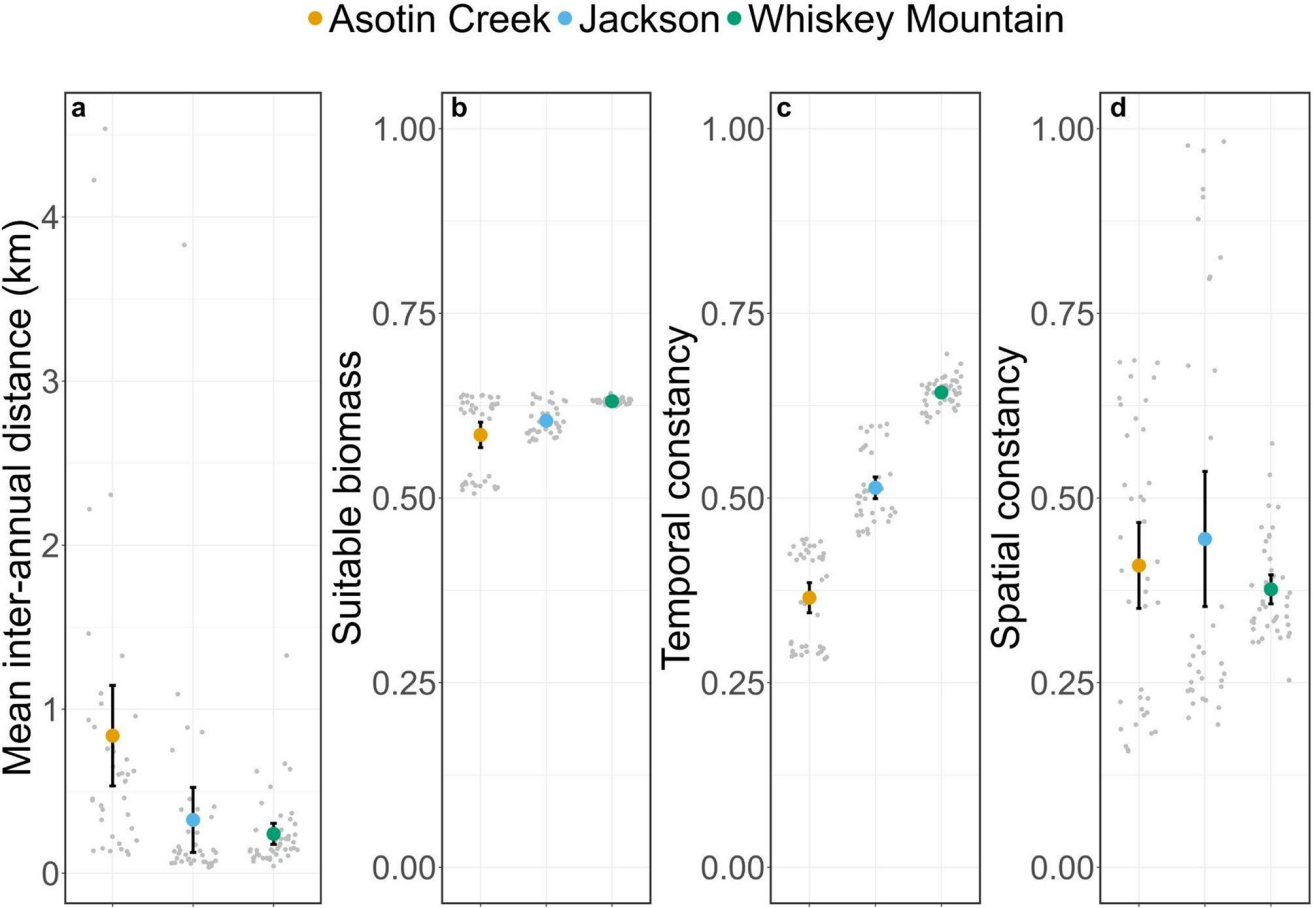
Mean (colored dots), 95% confidence intervals (black bars), and raw data (grey dots) for (**a**) inter-annual distance (i.e., site fidelity), (**b**) suitable biomass (i.e., home range quality), (**c**) temporal constancy, and (**d**) spatial constancy within summer home ranges of female bighorn sheep at Asotin Creek, Washington, USA (*n* = 12) and Jackson (*n* = 12) and Whiskey Mountain, Wyoming, USA (*n* = 17). Greater inter-annual distances correspond to lower levels of fidelity to inter-annual locations

**Fig. 4:**
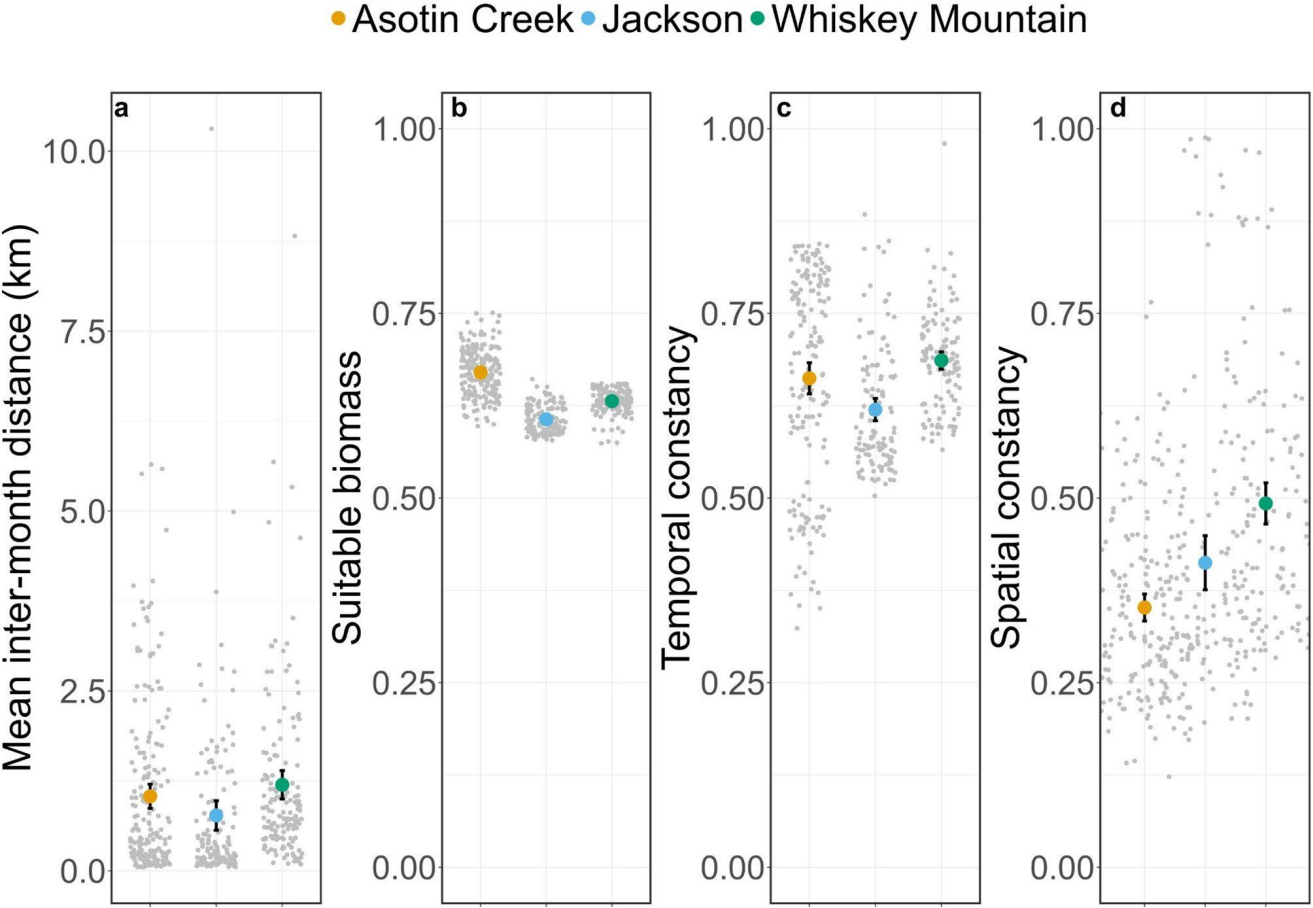
Mean (colored dots), 95% confidence intervals (black bars), and raw data (grey dots) for (**a**) inter-month distance (i.e., site fidelity), (**b**) suitable biomass (i.e., home range quality), (**c**) temporal constancy, and (**d**) spatial constancy within monthly home ranges of female bighorn sheep at Asotin Creek, Washington, USA (*n* = 25) and Jackson (*n* = 28) and Whiskey Mountain, Wyoming, USA (*n* = 29). Greater inter-month distances correspond to lower levels of fidelity to inter-month locations

**Fig. 5:**
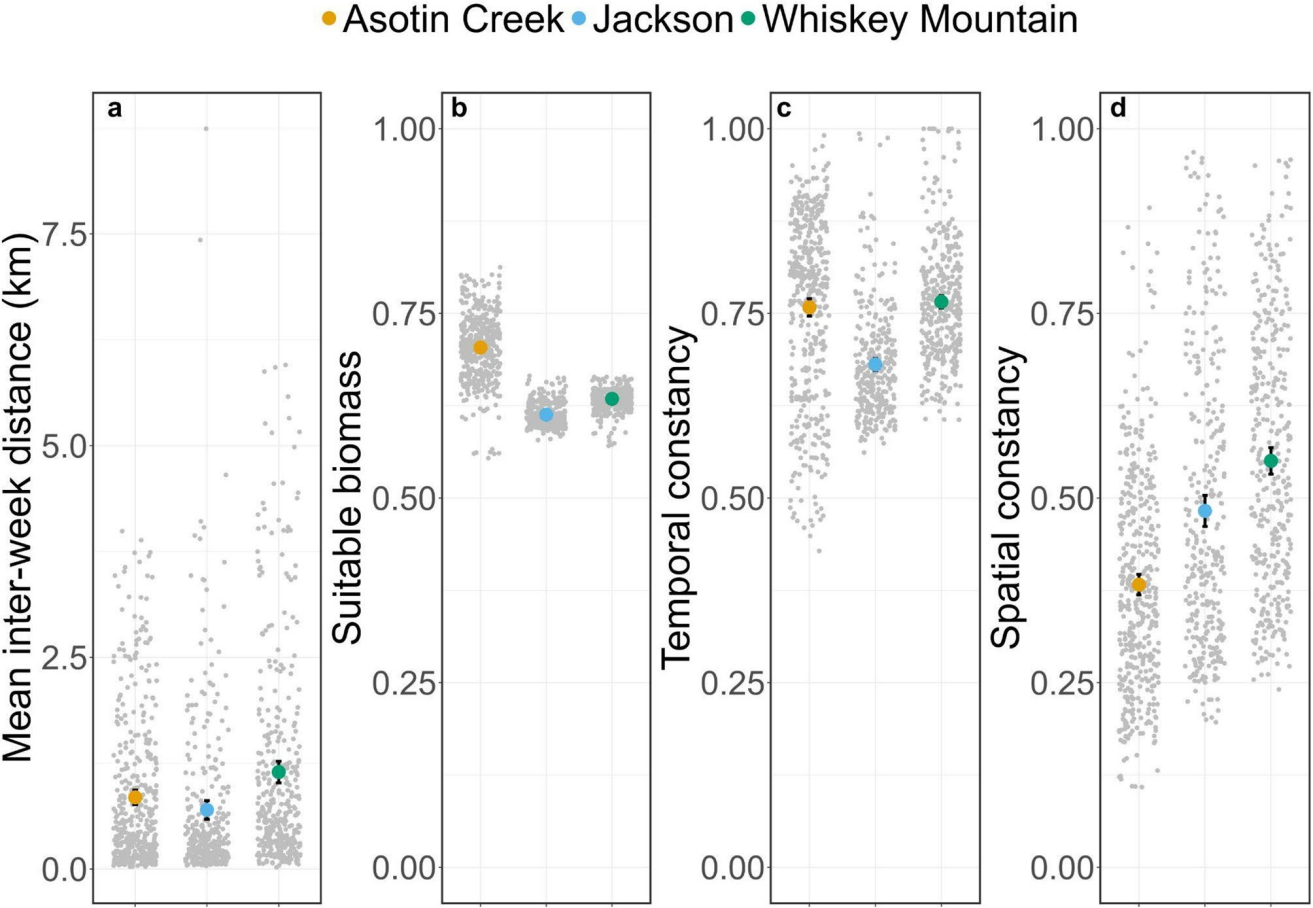
Mean (colored dots), 95% confidence intervals (black bars), and raw data (grey dots) for (**a**) inter-week distance (i.e., site fidelity), (**b**) suitable biomass (i.e., home range quality), (**c**) temporal constancy, and (**d**) spatial constancy within biweekly home ranges of female bighorn sheep at Asotin Creek, Washington, USA (*n* = 25) and Jackson (*n* = 28) and Whiskey Mountain, Wyoming, USA (*n* = 29). Greater inter-week distances correspond to lower levels of fidelity to inter-week locations

Site fidelity varied considerably among study areas and across temporal scales (Figs. 3, 4 and 5). Bighorn sheep at Asotin Creek (less temporally predictable grassland) were the least faithful (i.e., exhibited the greatest distance between GPS locations) at the inter-annual scale—mean inter-annual distance across individuals was 0.94 km compared to just 0.31 and 0.29 km in Jackson and Whiskey Mountain (more temporally predictable alpine), respectively (Fig. 3a). In contrast, site fidelity at the inter-month and inter-week scales was comparable among study populations—bighorn sheep were located an average of 300–800 m from sites used during the previous month or during the previous 14 days (Figs. 4a and 5a).

Environmental predictability and home-range quality varied among study areas and temporal scales. Forage resources across the Whiskey Mountain study area were generally predictable and high-quality across temporal scales (Additional File 3: Appendix 3; Figs. A1-3). Conversely, the Jackson study area contained forage resources that were more heterogeneous across space and time, and Asotin Creek was more heterogeneous across time, but the quality of forage resources within Jackson and Asotin Creek was comparable to Whiskey Mountain (Additional File 3: Appendix 3; Figs. A1-3). Environmental predictability and forage quality within home ranges occupied by bighorn sheep varied among study areas and temporal scales of analysis (Figs. 3, 4 and 5). Nevertheless, some general patterns were evident. Bighorn sheep at Jackson and Whiskey Mountain occupied home ranges that were generally predictable (with respect to suitable forage biomass) across time, whereas home ranges at Asotin Creek were temporally unstable (i.e., lower temporal constancy) at the inter-annual scale (Fig. 3). In contrast, home ranges of bighorn sheep at Whiskey Mountain were less spatially predictable (i.e., lower spatial constancy) than at either Jackson or Asotin Creek at the inter-annual scale (Fig. 3d). Bighorn sheep at Asotin Creek had the lowest-quality summer home ranges (i.e., lowest suitable biomass) of all three populations, and suitable biomass of forage was more temporally unpredictable (Figs. 3b, c). At the inter-month and inter-week scales, however, bighorn sheep at Asotin Creek had the highest-quality home ranges, and temporal predictability of suitable biomass increased (Figs. 4b-d and 5b-d). At Jackson and Whiskey Mountain, home-range quality was lowest at the inter-month and inter-week scales, respectively (Figs. 4b-d and 5b-d), and females occupied home ranges that were more predictable across space and time at the inter-month and inter-week scales (Figs. 4 and 5).

**Fig. 6:**
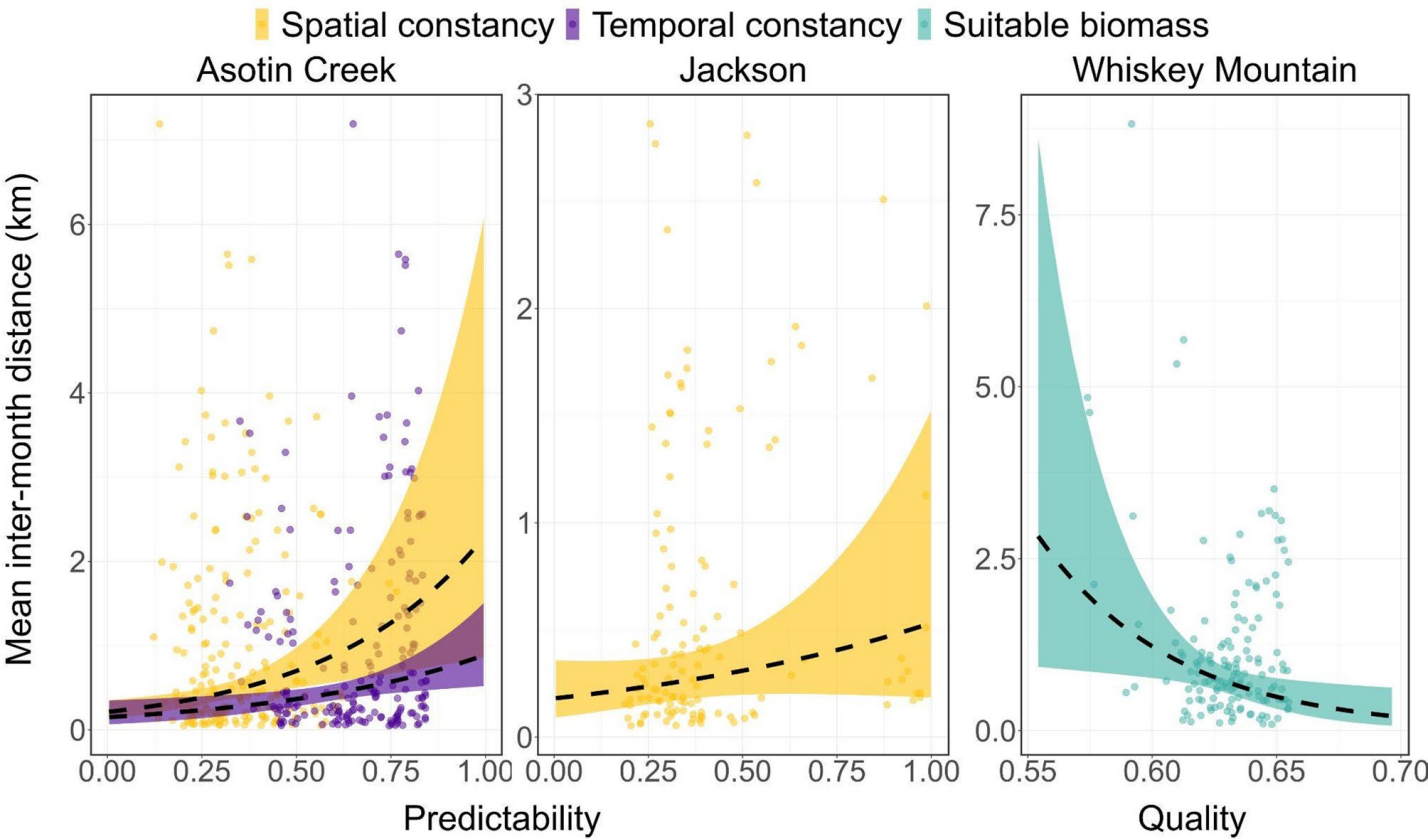
Model-predicted relationships (dashed lines), observed data (colored dots) and 95% confidence intervals (shaded regions) between monthly site fidelity (inter-month distance) and (1) environmental predictability (spatial and temporal constancy of the foodscape) and (2) home range quality (suitable biomass [kg ha^− 1^]; scaled between 0 and 1) of female bighorn sheep at Asotin Creek, Washington, USA and Jackson and Whiskey Mountain, Wyoming, USA

**Fig. 7:**
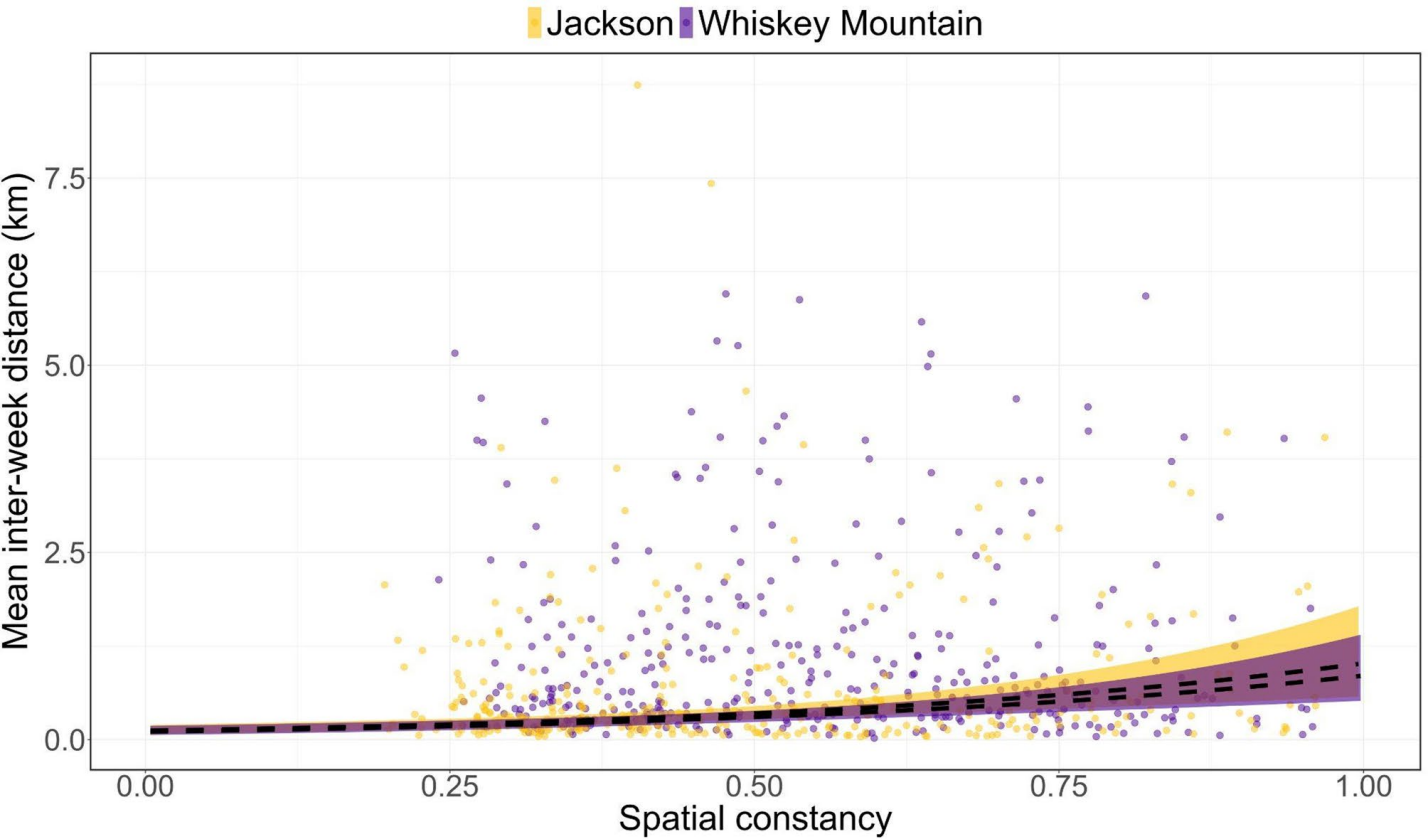
Model-predicted relationships (dashed lines) and 95% confidence intervals (shaded regions) between biweekly site fidelity (inter-week distance) and spatial constancy of the foodscape on site fidelity of female bighorn sheep at Jackson and Whiskey Mountain, Wyoming, USA

Despite their common occurrence in top model sets (Additional file 4: Appendix 4; Tables A1-3), neither environmental predictability nor home-range quality were useful predictors of inter-annual site fidelity by bighorn sheep at any study area (Tables 2, 3 and 4). Site fidelity also was unrelated to prior reproductive success across temporal scales. At the inter-month scale, metrics of environmental predictability were the only important determinants of site fidelity at Asotin Creek and Jackson, and metrics of quality (i.e., suitable biomass) determined inter-month site fidelity at Whiskey Mountain (Tables 2, 3 and 4). At Asotin Creek and Jackson, bighorn sheep that occupied spatially predictable home ranges were less faithful (i.e., higher inter-month distance) to sites visited in the previous month (Fig. 6; Tables 2, 3 and 4). Additionally, at Asotin Creek, bighorn sheep that occupied more temporally stable home ranges were less faithful to sites visited in the previous month (Fig. 6; Table 2). Conversely, bighorn sheep at Whiskey Mountain that experienced high-quality home ranges were more faithful (i.e., lower inter-month distance) to sites visited in the previous month (Fig. 6; Table 4). Environmental predictability and quality did not affect inter-week site fidelity of bighorn sheep at Asotin Creek (Table 2), but environmental predictability was an important determinant of inter-week site fidelity at Jackson and Whiskey Mountain (Tables 3 and 4)–site fidelity decreased (inter-week distance increased) as spatial predictability increased at both study sites (Fig. 7).

**Table 2:** Model coefficients across scales from Asotin Creek, Washington, USA

Temporal scale	Parameter	β	SE	Lower CI	Upper CI
Seasonal	Spatial constancy	0.16	0.14	-0.11	0.42
	Temporal constancy	-0.23	0.14	-0.49	0.04
	Suitable biomass	-0.14	0.14	-0.41	0.13
	Recruit	0.13	0.26	-0.37	0.63
	Home range size	0.17	0.15	-0.12	0.45
Monthly	**Spatial constancy**	0.29	0.09	0.12	0.47
	**Temporal constancy**	0.25	0.09	0.07	0.44
	Suitable biomass	-0.15	0.09	-0.33	0.04
	Recruit	0.24	0.18	-0.11	0.60
	**Home range size**	0.73	0.09	0.56	0.91
Biweekly	Spatial constancy	0.12	0.08	-0.03	0.28
	Temporal constancy	0.03	0.09	-0.14	0.20
	Suitable biomass	-0.06	0.08	-0.22	0.10
	Recruit	0.34	0.20	-0.05	0.73
	**Home range size**	0.59	0.09	0.41	0.76

Model-averaged coefficients (*β*), standard errors (*SE*), and 95% confidence intervals from linear mixed-effects models quantifying effects of environmental predictability (spatial and temporal constancy), home range quality (suitable biomass), recruitment success (recruit), and home range size (HR) on site fidelity of bighorn sheep at inter-annual, inter-month, and inter-week scales at Asotin Creek, Washington, USA. Bold values indicate significance (95% confidence intervals do not overlap zero). Recruit was a categorical variable, and coefficients represent contrasts between females that did or did not successfully recruit a neonate (unsuccessful females were used as the reference category)

**Table 3:** Model coefficients across scales from Jackson, Wyoming, USA

Temporal scale	Parameter	β	SE	Lower CI	Upper CI
Seasonal	Spatial constancy	0.18	0.24	-0.29	0.65
	Temporal constancy	0.36	0.24	-0.13	0.84
	Suitable biomass	-0.03	0.23	-0.48	0.41
	Recruit	-0.01	0.5	-0.99	0.97
	Home range size	0.41	0.23	-0.04	0.86
Monthly	**Spatial constancy**	0.27	0.11	0.05	0.49
	Temporal constancy	-0.15	0.11	-0.38	0.07
	Suitable biomass	-0.01	0.14	-0.29	0.27
	Recruit	0.44	0.24	-0.02	0.91
	**Home range size**	0.54	0.12	0.30	0.78
Biweekly	**Spatial constancy**	0.36	0.08	0.20	0.52
	Temporal constancy	-0.15	0.09	-0.32	0.02
	Suitable biomass	-0.08	0.10	-0.27	0.12
	Recruit	0.22	0.16	-0.10	0.54
	**Home range size**	0.67	0.08	0.51	0.83

Model-averaged coefficients (*β*), standard errors (*SE*) and 95% confidence intervals from linear mixed-effects models quantifying the effects of environmental predictability (spatial and temporal constancy), home range quality (suitable biomass), recruitment success (recruit), and home range size (HR) on site fidelity of bighorn sheep at inter-annual, inter-month, and inter-week scales at Jackson, Wyoming, USA. Bold values indicate significance (95% confidence intervals do not overlap zero). Recruit was a categorical variable, and coefficients represent contrasts between females that did or did not successfully recruit a neonate (unsuccessful females were used as the reference category)

**Table 4:** Model coefficients across scales from whiskey Mountain, Wyoming, USA

Temporal scale	Parameter	β	SE	Lower CI	Upper CI
Seasonal	Spatial constancy	-0.04	0.08	-0.21	0.12
	Temporal constancy	0.15	0.09	-0.02	0.31
	Suitable biomass	0.14	0.10	-0.05	0.33
	Recruit	0.31	0.28	-0.24	0.86
	**Home range size**	0.21	0.10	0.01	0.41
Monthly	Spatial constancy	0.19	0.13	-0.06	0.43
	Temporal constancy	-0.11	0.14	-0.38	0.15
	**Suitable biomass**	-0.31	0.11	-0.52	-0.10
	Recruit	0.33	0.18	-0.03	0.69
	**Home range size**	0.35	0.10	0.16	0.53
Biweekly	**Spatial constancy**	0.18	0.08	0.03	0.33
	Temporal constancy	-0.12	0.08	-0.28	0.03
	Suitable biomass	-0.06	0.07	-0.21	0.08
	Recruit	0.12	0.14	-0.15	0.39
	**Home range size**	0.62	0.07	0.49	0.75

Model-averaged coefficients (*β*), standard errors (*SE*)and 95% confidence intervals from linear mixed-effects models quantifying the effects of environmental predictability (spatial and temporal constancy), home range quality (suitable biomass), recruitment success (recruit), and home range size (HR) on site fidelity of bighorn sheep at inter-annual, inter-month, and inter-week scales at Whiskey Mountain, Wyoming, USA. Bold values indicate significance (95% confidence intervals do not overlap zero). Recruit was a categorical variable, and coefficients represent contrasts between females that did or did not successfully recruit a neonate (unsuccessful females were used as the reference category)

### Effects of site fidelity on individual performance

Mean ingesta-free body fat of female bighorn sheep in autumn was highest at Jackson ($\bar{x}$ = 14.10%, *SE =* 0.57) followed by Asotin Creek ($\bar{x}$ = 12.63%, *SE =* 0.37) and Whiskey Mountain ($\bar{x}$ = 12.00%, *SE =* 0.71). Juvenile survival to 120 days was generally high but varied considerably among years (50–90%) at Asotin Creek. Juvenile survival to 120 days at Jackson also was highly variable among years (25–78%), whereas survival was consistently low at Whiskey Mountain (11–17%). Site fidelity was not an important predictor of fat accrual or juvenile survival across most study areas and temporal scales of analysis (Table 5; see end of document). The only covariate that consistently influenced fat accumulation across populations and scales was recruitment status—females that successfully recruited a neonate entered winter in poorer condition and/or gained less fat over summer than females that did not (Table 5; see end of document). Indeed, recruitment status was 2–6 times more important as a predictor of fat gain over summer than site fidelity across all models (Table 5; see end of document). Additionally, females in the alpine study areas that exited winter with more fat accrued less fat over summer than females that exited winter with less fat (i.e., fat accrual was state dependent; Table 5; see end of document). Inter-annual site fidelity of maternal females did not differ between those with neonates that survived the summer and those that did not at Asotin Creek (*F* = -0.72, *P* = 0.47). Similarly, inter-annual site fidelity by females did not influence probability of neonate survival to 120 days at Jackson or Whiskey Mountain (*β*_*Jackson*_ = -0.53, *P*_*Jackson*_ = 0.22, *β*_*Whiskey Mountain*_ = 0.40, *P*_*Whiskey Mountain*_ = 0.12).

**Table 5:** Regression coefficients from linear mixed-effects models

Performance metric	Covariate	Inter-annual			Inter-month			Inter-week		
		*β*	*SE*	*P*	*β*	*SE*	*P*	*β*	*SE*	*P*
Autumn IFBFat	Fidelity	0.36	0.99	0.72	0.88	0.86	0.32	0.12	0.88	0.89
	Recruit	-2.61	1.45	**0.09**	-3.13	0.88	**< 0.01**	-3.07	0.91	**< 0.01**
	Fidelity: Recruit	0.07	1.38	0.95	-0.70	1.00	0.49	-0.39	1.02	0.70
Autumn IFBFat	Fidelity	1.27	1.33	0.35	-0.14	0.84	0.87	1.34	0.79	0.11
	Recruit	-3.47	1.76	**0.06**	-3.68	1.66	**0.03**	-4.35	1.29	**< 0.01**
	Fidelity: Recruit	-2.4	1.78	0.19	-0.85	1.78	0.63	-1.64	1.28	0.21
Percentage point gain	Fidelity	1.42	1.28	0.29	-0.25	0.86	0.77	1.31	0.81	0.11
	Spring fat	-4.72	0.87	**< 0.01**	-3.89	0.70	**< 0.01**	-3.72	0.69	**< 0.01**
	Recruit	-3.05	1.67	**0.09**	-3.59	1.63	**0.03**	-4.16	1.38	**< 0.01**
	Fidelity: Recruit	-2.94	1.71	0.11	-0.47	1.59	0.77	-1.45	1.33	0.28
Autumn IFBFat	Fidelity	1.02	0.71	0.17	-0.95	0.65	0.17	-0.35	0.70	0.63
	Recruit	-12.31	2.88	**< 0.01**	-10.89	2.78	**< 0.01**	-11.69	2.95	**< 0.01**
Percentage point gain	Fidelity	1.01	0.80	0.23	-0.93	0.68	0.19	-0.35	0.71	0.63
	Spring fat	-1.81	0.88	**0.07**	-2.33	0.71	**< 0.01**	-2.45	0.75	**< 0.01**
	Recruit	-12.34	3.41	**< 0.01**	-11.32	3.17	**< 0.01**	-12.34	3.26	**< 0.01**

Regression coefficients (*β*), *SEs*, and *P*-values from linear mixed-effects models used to evaluate the effects of inter-annual, inter-month, and inter-week site fidelity, recruitment status, and their interaction on performance (autumn nutritional condition [IFBFat] and, in the two alpine populations, percent change in body fat between spring and autumn) of female bighorn sheep at Asotin Creek, Washington, USA, and Jackson and Whiskey Mountain, Wyoming, USA, 2019–2022. Recruit was a categorical variable, and coefficients represent contrasts between females that did or did not successfully recruit a neonate into the population (unsuccessful females were used as the reference category). Bold font indicates *P* < 0.05

## Discussion

Despite the scale-dependent complexity of animal movements, many species have a strong tendency to revisit sites across years [9, 12, 84, 85]. Yet, comparative analyses of site fidelity within and among species are rare, making it challenging to uncover generalizable mechanisms underpinning the causes and consequences of this behavior [9, 86–88]. We examined the relative contributions of foodscape (suitable forage biomass) quality and predictability (i.e., public knowledge) and reproductive success (i.e., private knowledge) to site fidelity of bighorn sheep across multiple temporal scales. Site fidelity of bighorn sheep in our study areas (300–800 m; Figs. 3, 4 and 5) was higher than site fidelity estimates reported for many other ungulates (except mule deer and moose) but was comparable to other bighorn sheep populations [9]. Nevertheless, we found variable context-dependent relationships between foodscape conditions (i.e., forage predictability and quality within home ranges) and site fidelity, and no effect of reproductive success. Our results suggest that the strength and nature of site fidelity can vary considerably even within a species. Moreover, variable relationships between site fidelity and performance metrics among study areas and temporal scales suggest that the value of site fidelity also is context-dependent [89–91].

Animal movement is a scale-dependent process [30, 92–94], with the establishment of home ranges within a landscape and the choice of specific food items representing opposing ends of a continuum [30, 95]. Consequently, attempting to make inferences from animal movement patterns without considering the effects of spatial and temporal scale could lead to erroneous conclusions about the mechanisms underlying observed patterns, and ultimately how movement affects fitness [30]. Bighorn sheep were relatively faithful to previously visited sites at all temporal scales, with one notable exception—female sheep in the temporally less predictable grassland environment (Asotin Creek) were less faithful at the inter-annual scale than sheep inhabiting the temporally more predictable alpine environments (Whiskey Mountain and Jackson) (Fig. 4a). Suitable biomass at Asotin Creek was less predictable across space and time on the inter-annual scale than at either Jackson or Whiskey Mountain (Fig. 4). The Asotin Creek study area experienced a large-scale wildfire in July of 2021 that burned nearly 95% of the bighorn sheep summer range and resulted in a drastic decline in suitable biomass for the remainder of the summer (Additional File 3: Appendix 4; Fig. A4). This wildfire and subsequent loss of forage likely contributed to the low predictability of forage that we observed at Asotin Creek at the inter-annual scale. Indeed, forage quality at Asotin Creek was lower in 2021 compared to 2020 and 2022 and similarly, temporal and spatial predictability of forage were also lower during 2021, especially at the inter-month and inter-week scales (Additional File 3: Appendix 3; Figs. A5-7). The higher degree of foodscape heterogeneity at Asotin Creek—and the corresponding lower degree of predictability—likely caused bighorn sheep at that site to adopt a more nomadic movement strategy, leading to low inter-annual site fidelity during the study [92, 93, 95]. Similarly, roe deer (*Capreolus capreolus*) adopted a nomadic movement tactic during winter in unpredictable environments [92], and movement patterns of Mongolian gazelles (*Procapra gutturosa*) lacked regularity when spatiotemporal variation in vegetation greenness was high [96].

We observed substantial variation in both the quality and predictability of home ranges used by bighorn sheep across temporal scales (Figs. 3, 4 and 5). At the scale of the study area, spatial and temporal predictability of forage were generally lower than what we measured within individual bighorn sheep home ranges (Additional File 3: Appendix 3; Figs. A1-3), suggesting that sheep located their home ranges in areas that provided more predictable, high-quality forage. In addition, the relative importance of spatial versus temporal predictability in shaping behavior was evident in how bighorn sheep utilized home ranges over time. Forage resources within home ranges became more temporally stable as the time window narrowed from the inter-annual scale to the inter-month and inter-week scales. Similarly, spatial variation of forage within home ranges increased as the temporal window increased, but was more stable across time. While expected, this pattern reinforces the idea that temporal predictability is likely an important driver of site fidelity at broader temporal scales (e.g., years), whereas spatial predictability influences site fidelity more strongly at finer scales (e.g., months and weeks).

Bighorn sheep in alpine environments returned to sites visited during the previous year, but they did so regardless of their experience (i.e., whether they recruited a neonate) at those sites the previous year. Bighorn sheep at Asotin Creek were less faithful to previously visited sites at the inter-annual scale than alpine sheep. One explanation for the more rigid site fidelity exhibited by alpine sheep is a stronger reliance on memory for navigation [97–99]. Bighorn sheep in our alpine study areas were elevational migrants [39], and spatial memory is critical for migratory populations to consistently and efficiently access seasonal ranges from year to year [99]. Indeed, spatial memory had a remarkable influence on migration of mule deer (*Odocoileus hemionus*) in Wyoming and was 2–28 times more influential than spring green-up for determining fidelity to migration routes [99]. In contrast, bighorn sheep at Asotin Creek are non-migratory, and occupy the same relatively small range all year. Thus, individuals may be able to rely more heavily on real-time perceptual cues to optimize their use of forage resources [99, 100].

At fine temporal scales (i.e., inter-month and inter-week), site fidelity was variable among populations, and bighorn sheep responded differently to home-range predictability and quality (Figs. 4 and 5). We expected that suitable biomass in the low-elevation grasslands of Asotin Creek would be less predictable through time than in the alpine, and that, consequently, site fidelity by grassland sheep would be governed mainly by home-range predictability (i.e., bighorn sheep occupying home ranges with a more predictable forage base would exhibit greater site fidelity). Conversely, we predicted that suitable biomass in alpine environments would be more spatiotemporally predictable, and thus site fidelity would be governed primarily by previous reproductive success (i.e., win-stay, lose-switch; WSLS). Our predictions were only partially supported: site fidelity by bighorn sheep was related to home-range predictability and quality, but there was no effect of reproductive success on site fidelity in alpine bighorn sheep (Figs. 6 and 7; Tables 2, 3 and 4). The persistent effects of home-range quality and predictability on site fidelity suggest that cues associated with forage availability play a more important role in shaping movement strategies than previous reproductive success. Although the WSLS strategy is hypothesized to be beneficial for many species in predictable environments [87, 101], the foodscape in our study areas may not have been sufficiently homogeneous in space or time to promote site fidelity based solely upon past success (e.g [102]). We also acknowledge that our models of site fidelity did not account for predation risk. Predation risk is known to influence site fidelity across spatial and temporal scales [12, 19, 103], and may help to explain the limited effects of environmental predictability on site fidelity observed in our study. Incorporating both forage resources and predation risk into future models will provide a more comprehensive understanding of the factors shaping site fidelity and their consequences across temporal scales.

An alternative explanation for the absence of a relationship between prior reproductive success and site fidelity is that the metric we used to define reproductive success (i.e., juvenile survived the previous time interval) was not the fitness correlate most strongly driving site fidelity. Other currencies, such as foraging efficiency or net energy intake [89, 90], may have been more predictive of site fidelity than reproductive success. In long-lived herbivores, females should favor their own survival over current reproductive investment [104–107]. Body reserves represent the balance of energy intake and expenditure [53, 108, 109], and vital rates of ungulates, including adult survival, are strongly linked to nutrition [54]. This suggests that nutritional condition or fat gain could be better metrics for understanding the causes and consequences of site fidelity in long-lived herbivores. Although we did have estimates of seasonal variation in energy reserves (i.e., autumn IFBFat or percentage point gain), we did not obtain those data at finer temporal scales, and thus we were unable to quantify the effects of energy reserves on site fidelity across scales. Future research that explicitly measures net energy gain at fine temporal scales may shed additional light on factors shaping patterns of site fidelity in large herbivores.

Whiskey Mountain was the only study area in which the quality of home ranges (i.e., mean suitable biomass) influenced site fidelity—bighorn sheep that occupied higher-quality home ranges showed higher site fidelity at inter-month time scales than bighorn sheep that occupied lower-quality home ranges (Fig. 6; Table 4). In high-quality foraging habitat, animals may not have to move as far or as often to garner the resources they need to support survival and reproduction, leading to a positive relationship between habitat quality and site fidelity. For example, little penguins (*Eudyptula minor*) increased site fidelity when they experienced higher-quality foraging habitat during previous foraging bouts [86]. Similarly, moose that experienced declines in browse quality during winter were less faithful to previously visited sites during consecutive foraging intervals [30]. Bighorn sheep at Whiskey Mountain experienced the most stable foraging environment of all three populations—spatial and temporal predictability of forage was relatively high across temporal scales. Taken together, these results suggest that cues associated with forage quality may be important for determining site fidelity in more stable (i.e., more predictable) environments.

Numerous examples of variation in site fidelity exist across a wide range of taxa and scales (e.g [9, 88, 109]). Yet, very few studies have evaluated the performance consequences of site fidelity in free-ranging animals, primarily because of the difficulty of obtaining paired measurements of fidelity and performance (but see [86, 90]). We evaluated the effects of site fidelity on metrics of energy reserves (i.e., autumn nutritional condition or percentage point gain) and neonate survival to 120 days. Although we expected the performance metric most closely linked to site fidelity to differ between grassland and alpine environments, we nevertheless predicted that site fidelity would positively impact performance (i.e., body fat and nutritional condition survival), owing to the benefits of having reliable information about resource distribution and predation risk [8, 101, 103]. None of our results, however, supported this prediction. What we found instead was that recruitment status was 2–6 times more important than site fidelity for predicting variation in energy reserves across temporal scales and study areas (Table 5; see end of document). Females that incurred the costs of lactation were in consistently poorer condition in autumn and/or gained less fat over summer than females that did not recruit (Table 5; see end of document), independent of the strength of site fidelity. These results are a striking example of the fundamental life-history tradeoff wherein animals must choose between allocating energy to survival (i.e., accumulation of somatic reserves) versus reproduction [104]. Moreover, they suggest that standard metrics of site fidelity may not be sufficient for capturing fine-scale foraging behaviors employed by herbivores to maximize net energy gain.

Site fidelity at the inter-annual scale did not influence the probability of neonate survival to 120 days in any of our study areas. The lack of a relationship observed at Asotin Creek may be due to the cause of neonate mortalities–50% (3 of 6) of neonate mortalities were caused by accidents and trauma-related injuries (e.g., collisions with vehicles), which ostensibly occurred at random and were independent of maternal site fidelity. One additional explanation for the lack of a relationship among site fidelity and neonate survival is that rather than site fidelity influencing neonate survival, neonates impose limitations on space use of maternal females, and ultimately, site fidelity. For instance, a study conducted by Wagler et al. [110] in the same alpine study areas as ours found that overlap of consecutive biweekly home ranges used by female bighorn sheep decreased after loss of their neonate. An alternative explanation, perhaps, involves state-dependent site fidelity. In addition to increasing foraging efficiency, site fidelity is hypothesized to reduce predation risk. However, animals in poor condition may be more likely to take risks and to explore new locations to acquire resources [111, 112]. Bighorn sheep at Whiskey Mountain were, on average, in poorer condition than bighorn sheep in the other two populations, which could have led to more frequent exploratory movements [110]. Whereas we focused on condition as a consequence of site fidelity, future work should explore the hypothesis that site fidelity varies in response to nutritional condition and/or other state variables like disease status [113, 114].

Under some conditions, site fidelity also may be maladaptive. For example, strong fidelity to breeding sites by Baltic eiders (*Somateria mollissima*) resulted in drastic declines in the population due to high predation on adults by white-tailed sea eagles (*Haliaeetus albicilla*) [115]. Understanding the mechanisms that render site fidelity a maladaptive strategy is currently one of the most pressing questions in movement ecology, and future work focused on revealing these mechanisms will be important for predicting species’ responses to environmental variation, anthropogenic or otherwise [18].

## Conclusions

Human-induced environmental change, including marked shifts in plant phenology and extreme variation in climate, is the defining feature of the Anthropocene [18, 116, 117]. Understanding how animals adjust to rapid changes in their environment will be critical for forecasting and mitigating the effects of anthropogenic change on animal populations. Site fidelity is a behavioral mechanism that evolved in predictable resource environments, but that could become maladaptive in a rapidly changing world. Our study revealed that site fidelity of bighorn sheep is influenced by spatial and temporal variation in forage availability, suggesting that bighorn sheep have some flexibility to adjust to environmental change. Bighorn sheep inhabiting a grassland environment were flexible in their ability to shift behavior in response to changes in resource availability. However, there was little benefit to performance, indicating that site fidelity should only be maintained under certain conditions. Although bighorn sheep exhibited relatively strong site fidelity, our results suggest that land-use practices should aim to provide adequate space for bighorn sheep to shift space use in response to environmental change. Future work designed to sort out the conditions under which site fidelity is beneficial versus detrimental, and the capacity, behavioral or adaptive, of species to adjust patterns of site fidelity in response to environmental change, will shed important light on the mechanisms by which environmental variation influences individual and population performance.

## Supplementary Information

Below is the link to the electronic supplementary material.

Supplementary Material 1

Supplementary Material 2

Supplementary Material 3

Supplementary Material 4

## Data Availability

The datasets used and analyzed during this study will be available from the corresponding author on reasonable request.

## Abbreviations

WSLS: Win-stay: lose-switch
