# Supplementary material for: Should I stay or should I go? Causes and consequences of intraspecific variation in site fidelity
Source: Mov Ecol. 2025 Nov 6;13:80. doi: 10.1186/s40462-025-00606-w (PMC12590834; doi:10.1186/s40462-025-00606-w)
Supplement: Supplementary file 1 — Supplementary Material 1 [file 40462_2025_606_MOESM1_ESM.docx]

**Additional file 1: Appendix 1.** Methods for modeling spatiotemporal variation in suitable forage biomass (i.e., the foodscape) available to bighorn sheep at Asotin Creek, Washington, USA, and Jackson and Whiskey Mountain, Wyoming, USA.

**Estimating forage biomass**

During spring and summer, 2021–2022, we conducted intensive vegetation sampling to quantify spatiotemporal variation in energy and protein available to bighorn sheep in the Asotin Creek, Jackson, and Whiskey Mountain herds. We sampled vegetation from April–September, 2021–2022, in Asotin Creek, from June–September, 2021, in Whiskey Mountain, and from June–September, 2022, in Jackson (alpine study areas were inaccessible before June). We used the 30-m USDA Landfire Biophysical Settings Potential Vegetation Type (PVT) layer to stratify each study area into similar vegetation communities for sampling. The PVTs that composed each study area varied considerably–Asotin Creek was comprised primarily of the grassland community whereas conifers composed >50% of the Jackson study area. Whiskey Mountain was comprised of roughly equal portions of conifer, grassland, and sparsely vegetated communities (Table A1). Within each PVT we used Generalized Random Tessellation Stratified (GRTS) sampling in the ‘spsurvey’ package in Program R version 4.3.0 [1] to distribute transect locations in proportion to the relative area of the PVT within each study area [2].

At each sampling location, we placed a 100-m long transect that included 1-m^2^ quadrats every 20 m, beginning at 20 m and ending at 100 m. Within each quadrat, we identified all plants to species and phenological stage (i.e., new, flowering, fruiting, mature, cured, old). We estimated biomass of both green (i.e., live) and brown (i.e., dead/old growth) forage using a double sampling approach [2,3]. We first visually estimated percent horizontal cover (% cover) of each combination of species, part (current annual growth of stems versus leaves for shrubs, top 1/3 (leaves and inflorescence) versus bottom 2/3 (stems) in perennial forbs), and phenological stage in all 1-m^2^ quadrats. We then selected the two quadrats with the greatest diversity (i.e., total number of species) and used clip-and-weigh methods to estimate dry biomass of each unique combination of species, part, and phenological stage [3–5]. We clipped all species in those two quadrats at ground level up to 1.5 m (approximate maximum foraging height for bighorn sheep). After separating clipped samples by parts where necessary, we placed them into paper bags and dried them in a forced-air convection oven at 100°C for 24 h or until constant weight was achieved. After drying, we weighed samples using a platform scale (± 0.1 g), and any samples weighing ≤ 0.01 g were assigned a value of 0.01 g.

We used simple linear regression [6] to fit species-specific predictive models for estimating forage biomass in all unclipped quadrats as a function of % horizontal cover, Julian day, and the interaction between those variables [7]. We also evaluated natural log-transformed and quadratic terms for each predictor variable to test for potential non-linear relationships between covariates and forage biomass. We included tree canopy cover as a covariate in the biomass regressions for Jackson and Whiskey Mountain but did not include canopy cover in the models for Asotin Creek because 100 of 109 transects had 0% canopy cover, and average canopy cover was only 23% across transects that did include trees. We fit up to 14 competing models for each plant species for which we obtained ≥10 paired biomass and % horizontal cover measurements (Table A2). Some (16–27%) of our plant species had consistently low (i.e., <1%) % horizontal cover values, precluding meaningful regression analysis. In those instances we (1) calculated mean biomass of the species in all clipped quadrats where % cover was ≤1%, (2) assigned that mean biomass value to all unclipped quadrats where % cover was ≤1%, and (3) upscaled (linearly) the average biomass value to unclipped quadrats where % cover was >1% (e.g., for a quadrat with 2% cover we multiplied average biomass by 2 and assigned the resulting value to the quadrat; [5]). We assigned species with <10 paired biomass and % cover measurements to a functional group (evergreen shrubs, annual graminoids, perennial graminoids, perennial forbs, cacti, deciduous shrubs, deciduous trees, or conifers) prior to fitting biomass regressions. We set the intercept of all biomass regressions equal to 0 (i.e., regression through the origin;[5]). Once the respective species- or group-specific biomass regressions were applied to all unclipped quadrats, final biomass (kg ha^-1^) of each plant species at each transect location was calculated as the average biomass of the species across all five quadrats.

**Estimating forage quality**

For each unique combination of species, plant part, and phenological stage detected along a transect, we filled a quart-size Ziploc bag for analyses of nutritional quality and froze them at -18° C until the end of the field season. We then freeze dried all forb and shrub samples and air-dried grass and sedge samples in a paper bag at 40° C in a forced air convection oven for 24 h. We ground dried samples using a Wiley Mill with a 1-mm screen. We analyzed all nutrition samples collected in 2021 and 2022 for neutral detergent fiber (NDF, %), acid detergent lignin (ADL, %), and acid insoluble ash (AIA, %) sequentially using filter bags, alpha amylase, and the Ankom Fiber Analyzer ^200/220®^ (Ankom Technology, Fairport, NY, USA; [8]). We added 20 g of sodium sulfite to the ANKOM fiber analyzer at the NDF stage for all plant samples that potentially contained tannins. Graminoids do not have tannins and were assayed without sodium sulfite [9–11]. Sequential fiber analyses were conducted at the Wildlife Habitat Nutrition Laboratory at Washington State University (WSU) or the Idaho Department of Fish and Game Wildlife Health Laboratory (WHL). We corrected NDF, ADL, and AIA values using equations developed for that purpose by Cook et al. [10]:

$$CorrectedNDF=1.03334\times NDF+4.84719 (Eq. 1)$$

$$CorrectedADL=1.14617\times ADL+1.34973 (Eq. 2)$$

$$CorrectedAIA=2.869\times AIA-0.35145 (Eq. 3)$$

We assayed each sample submitted to WHL in duplicate and samples submitted to WSU were run as singles. For each corrected fiber component (i.e., NDF, ADL, and AIA) we took the average across duplicates and used the averages to calculate dry-matter digestibility (DMD, %; described below). If the difference in calculated DMD was > 2.5 percentage points, WHL analyzed a triplicate of the sample, and we averaged each corrected fiber component across all three samples before calculating DMD.

We analyzed a subset of nutritional quality samples collected in Jackson for gross energy (GE, kJ g^-1^) content using bomb calorimetry. Gross energy typically ranges from 17.6–20.1 kJ g^-1^, and a standard value of 18.8 kJ g^-1^ is often applied when study-specific estimates of GE are not available, which is often the case due to funding constraints (bomb calorimetry costs ~$33/sample) [11,12]. However, estimates of GE are lacking for plants in high alpine environments. Accordingly, we pooled nutrition samples collected in Jackson for GE analysis by genus and by temporal categories within each transect based on the time of collection (i.e., early vs. late in the sampling season). We randomly selected samples from three different transects to assay for GE (except for genera found on < 3 transects, in which case we submitted all samples) and estimated the mean and standard deviation of GE from those samples. We applied mean GE values from each unique combination of genus and time period to all corresponding samples collected in Jackson and Whiskey Mountain. Wagoner et al. [13] quantified GE of plants in Asotin Creek at the species level, and we used those authors’ values for plants in that study area.

We analyzed all samples collected in Asotin Creek and Whiskey Mountain in 2021 and in Jackson in 2022 for nitrogen content using the Dumas method and a Carbon–Nitrogen TruSpec analyzer (LECO, St. Joseph, MI, USA) at Dairy One Forage Laboratory, Ithaca, NY, USA. We estimated crude protein content (CP, %) as 6.25 × the nitrogen content [12]. Due to budgetary constraints during 2022, we pooled the >900 nutrition samples collected in Asotin Creek for CP analysis by species, plant part, and phenological stage. For each combination of species, plant part, and phenological stage, we randomly partitioned the samples into three groups. We then selected three samples (~0.5 g each) from each group at random and pooled them into a single sample to assay. We did not pool samples for species-part-phenological stages with *n* < 9. For pooled samples, the mean CP value was assumed to be representative of all transects that included that species-part-phenological stage combination.

Secondary metabolites, especially tannins, can limit protein absorption by animals [14]. Accordingly, we estimated protein-precipitating capacity of condensed tannins (mg Bovine Serum Albumin precipitate/mg forage; [15]) for a subset of forage samples collected in Asotin Creek (2021 and 2022) and Whiskey Mountain (2021); we excluded Jackson samples from tannin analysis because vegetation communities in that study area were very similar to those at Whiskey Mountain. Grasses, sedges, and plants in the *Asteraceae* and *Liliaceae* families typically do not contain tannins [10], and thus we excluded those taxa from tannin analysis. We pooled the remaining forb and shrub samples by species, plant part, and phenological stage within each study area. Tannin analysis is costly (~$50/sample), and we attempted to minimize those costs by conducting tannin analyses in two stages. We began by assaying the earliest phenological stage sampled (i.e., the time when tannins are most likely to be present) for each species-plant part combination within the Asotin Creek and Whiskey Mountain study areas. Although canopy cover is a primary driver of intraspecific variation in tannins, our study areas consisted largely of open, rocky habitats, and thus we did not stratify plant samples by canopy cover for tannin analysis. If a sample from the first stage of analysis precipitated protein (mg BSA/mg forage > 0, hereafter contained tannins), we proceeded by analyzing one randomly selected sample from each successive phenological stage of that species-plant part combination. For species that did not contain tannins in the first stage of analysis, no further tannin analyses were conducted. Tannin values for plant species sampled at Whiskey Mountain were applied to all matching species-plant part-phenological stages sampled in Jackson. For species that were sampled in Jackson but not in Whiskey Mountain (*n* = 157), we applied the mean tannin value of the corresponding genus and phenological stage from Whiskey Mountain. If there were no matching genera, we applied the mean value for the corresponding family and phenological stage. All tannin analyses were conducted at the Wildlife Habitat Nutrition Laboratory at Washington State University. A summary of forage quality analyses conducted for each study area is provided in Table A3.

We estimated DMD for each plant sample by entering estimates of NDF, ADL, AIA, and BSA into the summative equations of Robbins et al. [14]. The equations were initially developed for deer and elk; however, we assumed that the relationship would be similar for bighorn sheep. We then calculated the digestible energy (DE) content (kJ g^-1^) of each forage sample as the product of GE and DMD. We calculated digestible protein (DP) content (g protein/100 g forage) of each forage sample by entering estimates of CP and BSA into the corresponding equation from Robbins et al. [14]. We used DE and DP as our estimates of forage quality in all subsequent analyses.

**Estimating suitable forage biomass**

We entered data on forage biomass and quality from each sampling location (i.e., transect) into the Forage Resource Evaluation System for Habitat (FRESH) model [11], a linear programming framework that estimates suitable forage biomass, defined as the maximum biomass of available forage that, pooled together, meets nutritional requirements (DE and DP) for supporting a specified level of performance (e.g., lactation) of the modeled herbivore. We partitioned the biomass of all plant taxa by plant part, and entered corresponding estimates of DE ± *SD* and DP ± *SD* into the FRESH model. To account for variation in nutritional quality within plant species and part, we assigned 1/3 of the biomass of each species and part to its mean DE and DP values, 1/3 to the mean plus 1 SD, and 1/3 to the mean minus 1 SD [11,16]. We did not allow evergreen shrubs (*Artemisia* spp., *Chrysothamnus* spp., *Ericameria* spp.) or *Lupinus* spp. to contribute to modeled diets because although these taxa are high in digestible protein, bighorn sheep typically avoid consuming them during the summer months, ostensibly due to high concentrations of plant secondary metabolites [17,18]. The nutritional requirements of lactating bighorn sheep have not been reported, and thus we estimated requirements for DE and DP based on published values for wild (i.e., mule deer [13] and elk [19,20]) and domestic (i.e., sheep [*Ovis aries;* 21]) ruminants of similar size and life history traits. For a 70-kg lactating female bighorn sheep, we assumed a DE requirement of 11.5 kJ g^-1^ and a DP requirement of 7.5 g/100 g forage [14].

**Modeling the foodscape**

We used generalized additive models (GAMs; [22]) to quantify spatiotemporal variation in the foodscapes (i.e., suitable forage biomass) available to bighorn sheep in the Asotin Creek, Jackson, and Whiskey Mountain populations during spring and summer, 2021–2022 [2]. We used estimates of suitable biomass at each transect location as the response variable and evaluated the predictive value of numerous spatial and temporal covariates for each study area and year. We conducted model selection in two stages to (1) assess which covariates to retain, and (2) optimize functional form of the relationship between each covariate and suitable biomass [2,5]. Our primary goal was to maximize the predictive strength of each model rather than to make inferences from model coefficients. We began the first stage of model fitting by evaluating all possible combinations of spatial (x-y coordinates) and temporal (Julian day, maximum temperature, mean temperature [since March 1], cumulative precipitation [since March 1], soil moisture, solar radiation, NDVI, and EVI) smoothing terms. We selected the model with the most predictive combination of smoothing terms based on adjusted *R^2^* values. We also log-transformed suitable biomass in the Asotin Creek model to ensure normality and adherence to model assumptions. We then fit a series of models that included the optimal combination of smoothing terms along with a single fixed effect from a list of candidate covariates; candidate spatial and temporal covariates are shown in Table A4. For pairs of variables that were correlated (*r* |≥| 0.6), we used adjusted *R^2^* of the corresponding single-covariate models to determine which variable to retain. The resulting global model from the first stage of analysis contained all uncorrelated, linear fixed effects.

In the second stage of analysis, we iteratively evaluated five candidate models for each continuous covariate in the global model: (1) untransformed covariate; (2) covariate^2^, (3) covariate^3^, (4) ln(covariate), and (5) covariate removed. Each model specified a different functional form of the relationship between suitable biomass and the focal covariate, and we used adjusted *R^2^* values to determine which form of the covariate to retain. We fit models and assessed adherence of the final model to assumptions using the ‘mgcv’ package in Program R version 4.3.0 [23]. The top GAMs for each study area are presented in Table A5, and the coefficients from the top GAMs are presented in Table A6.

**REFERENCES**

1. Dumelle M, Kincaid T, Olsen AR, Weber M. spsurvey: Spatial sampling design and analysis in R. J Stat Softw. 2023;105:1–29.

2. Merems JL, Shipley LA, Levi T, Ruprecht J, Clark DA, Wisdom MJ, Jackson NJ, Stewart KM, Long RA. Nutritional-landscape models link habitat use to condition of mule Deer (Odocoileus hemionus). Front. Ecol. Evol. 2020;8 https://doi.org/10.3389/fevo.2020.00098

3. Bonham CD. Measurements for terrestrial vegetation. John Wiley and Sons; 2013.

4. Proffitt KM, Hebblewhite M, Peters W, Hupp N, Shamhart J. Linking landscape-scale differences in forage to ungulate nutritional ecology. Ecol Appl. 2016;26:2156–74.

5. Bilodeau N. Context-dependent effects of nutrition and dam behavior on neonatal survival in a long-lived herbivore. University of Idaho; 2021.

6. Neter J, Wasserman W, Kutner MH. Applied linear regression. Chic IL Irwin. 1996;720.

7. Monzingo DS, Shipley LA, Cook RC, Cook JG. Factors influencing predictions of understory vegetation biomass from visual cover estimates. Wildl Soc Bull. 2022;46:e1300.

8. Goering HK, Van Soest PJ. Forage fiber analysis (apparatus, reagents, procedures and some applications). U.S. Department of Agriculture Research Station. Agriculture Handbook 379. Government Printing Office, Washington, D.C., USA. 1970.

9. Mould ED, Robbins CT. Evaluation of detergent analysis in estimating nutritional value of browse. J Wild. Manag. 1981;45:937–947.

10. Cook RC, Shipley LA, Cook JG, Camp MJ, Monzingo DS, Robatcek SL,Berry SL, Hull IT, Myers WL, Denryter K, Long RA. Sequential detergent fiber assay results used for nutritional ecology research: Evidence of bias since 2012. Wildl Soc Bull. 2022;46:e1348.

11. Hanley TA, Spalinger DE, Mock KJ, Weaver OL, Harris GM. Forage resource evaluation system for habitat—deer: an interactive deer habitat model. Gen Tech Rep Portland US Dep Agric For Serv Pac Northwest Res Stn 2012;858. Available from: https://www.fs.usda.gov/research/treesearch/40300

12. Robbins CT. Wildlife feeding and nutrition. Elsevier;1993.

13. Wagoner SJ, Shipley LA, Cook RC, Hardesty L. Spring cattle grazing and mule deer nutrition in a bluebunch wheatgrass community. J Wildl Manag. 2013;77:897–907.

14. Robbins CT, Hanley TA, Hagerman AE, Hjeljord O, Baker DL, Schwartz CC, Mautz WW. Role of tannins in defending plants against ruminants: reduction in protein availability. Ecology. 1987;68:98–107.

15. Martin JS, Martin MM. Tannin assays in ecological studies: lack of correlation between phyenolics, proanthocyanidins and protein-precipitating constituents in mature foliage of six oak species. Oecologia. 1982;54:205–211.

16. Hull IT, Shipley LA, Berry SL, Loggers C, Johnson TR. Effects of fuel reductions timbers harvests on forage resources for deer in northeastern Washington. For. Ecol. Manag. 2020;458:e117757.

17. Cook JG. Habitat, nutrition, and population ecology of two transplanted bighorn sheep populations in southcentral Wyoming. University of Wyoming; 1990

18. Wagner G, Peek J. Bighorn sheep diet selection and forage quality in Central Idaho. Northwest Sci. 2006;80.

19. Cook JG, Johnson BK, Cook RC, Riggs RA, Delcurto T, Bryant LD, Irwin LL. Effects of summer-autumn nutrition and parturition date on reproduction and survival of elk. Wildl Monogr. 2004;155:1–61.

20. Monzingo DS, Cook JG, Cook RC, Horne JS, Shipley LA. Influences of succession and biogeoclimate on forage resources for elk in northern Idaho. Northwest Sci. 2023;96:94–116.

21. National Research Council. Nutrient requirements of small ruminants: sheep, goats, cervids, and new world camelids. The National Academies Press, Washington, D.C., USA. 2007.

22. Zuur AF, Ieno EN, Walker NJ, Saveliev AA, Smith GM. Mixed effects models and extensions in ecology with R. Springer; 2009

23. Wood SN. Generalized additive models: an introduction with R, 2nd Edition. CRC Press; 2017.

24. Abatzoglou JT, Dobrowski SZ, Parks SA, Hegewisch KC. Terraclimate, a high-resolution global dataset of monthly climate and climatic water balance from 1958-2015. Sci. Data. 2018; 5:e170191.

25. McCune B, Keon D. Equations for potential annual direct incident radiation and heat load. J Veg. Sci. 2002; 13: 603.

26. Riley SJ, DeGloria SD, Elliot R. A terrain ruggedness index that quantifies topographic heterogeneity. Inter. J Sci. 1999;5: 23–27.

Table A1. Potential vegetation types in Asotin Creek, Washington, USA, and Jackson and Whiskey Mountain, Wyoming, USA. Columns 3 and 4 indicate the percentage of (1) each population range, and (2) the GPS-location dataset obtained from collared sheep at each site, comprised of each PVT. Bold font denotes PVTs that were sampled to estimate forage biomass and quality within each study area.

| Study area | PVT | Percent pop. range | Percent locations |
| --- | --- | --- | --- |
| Asotin Creek | **Conifer** | 9.10 | 12.60 |
|  | **Grassland** | 80.65 | 81.82 |
|  | Hardwood | 0.08 | 0.06 |
|  | **Riparian** | 2.37 | 0.46 |
|  | **Shrubland** | 7.78 | 5.06 |
|  |  |  |  |
| Jackson | **Conifer** | 56.32 | 46.42 |
|  | **Grassland** | 5.47 | 9.66 |
|  | **Hardwood** | 7.19 | 6.57 |
|  | Riparian | 2.02 | 1.32 |
|  | **Shrubland** | 14.17 | 20.38 |
|  | **Sparse** | 12.36 | 14.21 |
|  |  |  |  |
| Whiskey Mountain | **Conifer** | 39.48 | 42.68 |
|  | Hardwood | 1.04 | 1.58 |
|  | Riparian | 1.02 | 1.46 |
|  | **Shrubland** | 28.68 | 24.41 |
|  | **Sparse** | 29.49 | 30.52 |

Table A2. Species-specific linear regressions of plant biomass against percent plant cover (%), sample date (i.e., Julian day [JD]), canopy cover [CC], and various transformations and interactions of those variables. Coefficients are shown for variables included in the best model for each species or growth-form group, along with the adjusted *R^2^* value of the model. Species for which it was not appropriate to fit a regression model were assigned a species-specific mean biomass value in the species cover column (see Methods). We used these models to estimate forage biomass in all unclipped quadrats sampled in each study area. For graminoids, we differentiated between brown and green biomass.

| Study area | Year | Latin name | *n* | Adj. *R^2^* | % cover | log(%cover) | JD | log(JD) | % cover:JD | log(% cover):log(JD) | JD^2^ | CC | % cover: CC | log(CC) |  |
| --- | --- | --- | --- | --- | --- | --- | --- | --- | --- | --- | --- | --- | --- | --- | --- |
|  |  |  |  |  |  |  |  |  |  |  |  |  |  |  |  |
| Asotin Creek | 2021 | *Achillea millefolium*-LVS | 17 | 0.82 | 6.84 |  | -0.02 |  |  |  |  |  |  |  |  |
|  |  | *Artemisia tridentata* | 26 | 0.92 |  | 5.47 |  | 0.09 |  | -0.90 |  |  |  |  |  |
|  |  | *Bromus japonicus*-BROWN | 22 | 0.71 | -13.44 |  | -0.05 |  | 0.23 |  |  |  |  |  |  |
|  |  | *Bromus japonicus*-GREEN | 31 | 0.80 | 2.86 |  | -0.05 |  |  |  | <0.01 |  |  |  |  |
|  |  | *Bromus* spp. | 39 | 0.94 | 2.52 |  | -0.02 |  |  |  | <0.01 |  |  |  |  |
|  |  | *Bromus tectorum*-BROWN | 20 | 0.76 | 3.52 |  | -0.05 |  |  |  | <0.01 |  |  |  |  |
|  |  | *Bromus tectorum*-GREEN | 37 | 0.81 | 2.86 |  | 0.01 |  |  |  |  |  |  |  |  |
|  |  | *Calachortus macrocarpus* | 11 | 0.78 |  | 55.29 |  | 7.49 |  |  |  |  |  |  |  |
|  |  | *Festuca idahoensis* | 15 | 0.98 |  | 5.74 |  | 0.17 |  | -0.91 |  |  |  |  |  |
|  |  | *Lactuca seriola* | 12 | 0.69 |  | 69.77 |  | -0.07 |  | -12.97 |  |  |  |  |  |
|  |  | *Phlox longifolia* | 16 | 0.46 | 2.48 |  |  |  |  |  |  |  |  |  |  |
|  |  | *Poa bulbosa* | 14 | 0.58 | 2.52 |  |  |  |  |  |  |  |  |  |  |
|  |  | *Poa secunda* | 46 | 0.77 | 2.35 |  | <0.01 |  | <0.01 |  |  |  |  |  |  |
|  |  | *Pseudoroegnaria spicata*-BROWN | 12 | 0.94 |  | -21.06 |  | 0.38 |  | 4.45 |  |  |  |  |  |
|  |  | *Pseudoroegnaria spicata*-GREEN | 53 | 0.94 |  | 2.78 |  | 0.29 |  | -0.32 |  |  |  |  |  |
|  |  | *Pseudoroegnaria spicata*-GREEN/BROWN | 21 | 0.97 |  | 1.10 |  | 0.29 |  |  |  |  |  |  |  |
|  |  | Perennial forbs | 103 | 0.62 |  | 5.75 |  | 0.09 |  | -0.84 |  |  |  |  |  |
|  |  | Annual forbs | 73 | 0.92 | 6.17 |  |  |  |  |  |  |  |  |  |  |
|  |  | Perennial graminoids | 16 | 0.91 |  | 3.80 |  | 0.24 |  | -0.54 |  |  |  |  |  |
|  |  | Annual graminoids | 10 | 0.89 |  | 59.5 |  | 0.19 |  | -11.08 |  |  |  |  |  |
|  |  | Evergreen shrubs | 11 | 0.93 | 2.10 |  |  |  |  |  |  |  |  |  |  |
|  |  | Deciduous shrubs | 12 | 0.98 | -4.23 |  | <0.01 |  | 0.03 |  |  |  |  |  |  |
|  |  | Cactus | 10 | 0.95 | -7.71 |  | -0.01 |  | 0.13 |  |  |  |  |  |  |
| Asotin Creek | 2022 | *Achillea millefolium*-LVS | 46 | 0.83 | -3.48 |  | -0.03 |  | 0.02 |  |  |  |  |  |  |
|  |  | *Achillea millefolium*-FLRS | 15 | 0.98 | 5.13 |  | -0.01 |  | <0.01 |  |  |  |  |  |  |
|  |  | *Apera interrupta* | 10 | 0.98 | -7.4 |  | -0.01 |  | 0.08 |  |  |  |  |  |  |
|  |  | *Bromus japonicus* | 59 | 0.76 |  | -17.5 |  | 0.18 |  | 3.71 |  |  |  |  |  |
|  |  | *Bromus tectorum* | 91 | 0.88 |  | -1.73 |  | 0.18 |  | 0.61 |  |  |  |  |  |
|  |  | *Chamaesyce glyptosperma* | 10 | 0.82 | -22.42 |  | -0.01 |  | 0.12 |  |  |  |  |  |  |
|  |  | *Decurainia incana* | 23 | 0.94 | 9.71 |  | <0.01 |  | -0.04 |  |  |  |  |  |  |
|  |  | *Epilobium brachycarpum* | 10 | 0.59 |  | 2.63 |  |  |  |  |  |  |  |  |  |
|  |  | *Erodium circutarium* | 14 | 0.85 | -1.19 |  | <0.01 |  | 0.02 |  |  |  |  |  |  |
|  |  | *Festuca idahoensis* | 11 | 0.95 |  | 0.58 |  | 0.29 |  |  |  |  |  |  |  |
|  |  | *Holosteum umbellatum* | 15 | 0.80 | 6.03 |  | <0.01 |  | -0.02 |  |  |  |  |  |  |
|  |  | *Lactuca seriola* | 35 | 0.77 |  | 27.31 |  | -0.08 |  | -4.73 |  |  |  |  |  |
|  |  | *Myosotis stricta* | 34 | 0.80 |  | -7.58 |  | 0.03 |  | 1.75 |  |  |  |  |  |
|  |  | *Phacelia heterophylla* | 23 | 0.84 | -0.29 | <0.01 |  | <0.01 |  |  |  |  |  |  |  |
|  |  | *Phacelia linearis* | 16 | 0.91 | 1.47 |  | -0.05 |  |  | <0.01 |  |  |  |  |  |
|  |  | *Phlox longifolia* | 10 | 0.75 |  | -33.90 |  | 0.03 |  | 6.96 |  |  |  |  |  |
|  |  | *Plantago patagonica* | 15 | 0.70 |  | 2.43 |  |  |  |  |  |  |  |  |  |
|  |  | *Poa bulbosa* | 28 | 0.91 | -1.49 |  | <0.01 |  | 0.03 |  |  |  |  |  |  |
|  |  | Sandberg bluegrass | 73 | 0.90 | 2.02 |  | <0.01 |  | 0.01 |  |  |  |  |  |  |
|  |  | *Pseudoroegnaria spicata*-BROWN | 18 | 0.93 |  | 1.11 |  | 0.23 |  |  |  |  |  |  |  |
|  |  | *Pseudoroegnaria spicata*-GREEN | 69 | 0.97 |  | -1.47 |  | 0.26 |  | 0.48 |  |  |  |  |  |
|  |  | *Sisymbrium altissimum* | 48 | 0.92 | -5.09 |  | 0.01 |  | 0.04 |  |  |  |  |  |  |
|  |  | *Vulpia octiflora* | 10 | 0.68 | 2.84 |  |  |  |  |  |  |  |  |  |  |
|  |  | Perennial forbs | 129 | 0.84 | 0.88 |  | <0.01 |  | <0.01 |  |  |  |  |  |  |
|  |  | Annual forbs | 112 | 0.74 | 2.23 |  | -0.03 |  |  | <0.01 |  |  |  |  |  |
|  |  | Perennial graminoids | 22 | 0.96 |  | 0.87 |  | 0.21 |  |  |  |  |  |  |  |
|  |  | Annual graminoids | 15 | 0.86 | -4.20 |  | <0.01 |  | 0.03 |  |  |  |  |  |  |
|  |  | Evergreen shrubs | 18 | 0.92 |  | -7.87 |  | 0.07 |  | 1.74 |  |  |  |  |  |
|  |  | Deciduous shrubs | 10 | 0.98 |  | 1.09 |  | 0.11 |  |  |  |  |  |  |  |
| Whiskey Mountain | 2021 | *Potentilla diversifolia* | 11 | 0.97 | -0.57 |  | <0.01 |  | <0.01 |  |  |  |  |  |  |
|  |  | *Phlox pulvinata* | 12 | 0.82 | 0.82 |  |  |  |  |  |  |  |  |  |  |
|  |  | *Koeleria macrantha* | 15 | 0.80 | 3.66 |  | -0.01 |  |  |  |  |  |  |  |  |
|  |  | *Astragalus miser* | 11 | 0.88 |  | 10.99 |  | 0.08 |  | -1.93 |  |  |  |  |  |
|  |  | *Artemisia tridentata* | 18 | 0.93 |  | 1.21 |  |  |  |  |  |  |  |  |  |
|  |  | *Pseudoroegnaria spicata* | 27 | 0.96 |  | -14.67 |  | 0.14 |  | 3.08 |  |  |  |  |  |
|  |  | *Poa secunda* | 17 | 0.73 | 1.86 |  | <0.01 |  |  |  |  | 1.07 | -0.49 |  |  |
|  |  | *Artemisia frigida* | 17 | 0.80 | 15.08 |  | <0.01 |  | -0.07 |  |  |  |  |  |  |
|  |  | *Tetraneuris acaulis* | 10 | 0.64 | -0.14 |  | 0.17 |  |  |  | <0.01 |  |  |  |  |
|  |  | Perennial graminoids | 66 | 0.80 | -0.21 |  | <0.01 |  | <0.01 |  |  |  |  |  |  |
|  |  | Perennial forbs | 142 | 0.81 | 1.91 |  | <0.01 |  |  |  |  | 0.04 |  | -0.02 |  |
|  |  | Subshrub | 12 | 0.72 | 7.42 |  | <0.01 |  | -0.03 |  |  |  |  |  |  |
|  |  |  | 10 | 0.96 |  | 1.42 |  | -0.22 |  |  |  |  |  | 0.08 |  |
| Jackson | 2022 | *Achillea millefolium* | 18 | 0.84 |  | 2.85 |  | -0.34 |  |  |  |  |  | 2.74 |  |
|  |  | *Arnica cordifolia* | 16 | 0.93 |  | 1.01 |  | 0.16 |  |  |  |  |  | 2.34 |  |
|  |  | *Artemisia tridentata* | 11 | 0.94 |  | 0.69 |  | 0.09 |  |  |  |  |  | 0.04 |  |
|  |  | *Astragalus miser* | 10 | 0.93 |  | 1.49 |  | -0.13 |  |  |  |  |  | <0.01 |  |
|  |  | *Elymus trachycaulus* | 11 | 0.92 | 1.94 |  | <0.01 |  |  |  |  | -0.30 | <0.01 |  |  |
|  |  | *Arenaria congesta* | 12 | 0.91 | 0.94 |  |  |  |  |  |  |  |  |  |  |
|  |  | *Erigeron leiomerus* | 11 | 0.71 | 1.64 |  | <0.01 |  |  |  |  | 0.02 |  | -0.02 |  |
|  |  | *Leukopoa kingii* | 13 | 0.96 | -0.28 |  | <0.01 |  | <0.01 |  |  |  |  |  |  |
|  |  | *Paxistima myrsinites* | 12 | 0.96 | -0.14 |  | -0.01 |  | <0.01 |  |  |  |  |  |  |
|  |  | *Phlox hoodii* | 11 | 0.92 |  | 1.32 |  | -0.05 |  |  |  |  |  |  |  |
|  |  | *Poa secunda* | 13 | 0.63 |  | 18.94 |  | 0.09 |  | -3.47 |  |  |  |  |  |
|  |  | *Solidago multiradiata* | 19 | 0.93 |  | 9.78 |  | -0.03 |  | -1.66 |  |  |  |  |  |
|  |  | *Taraxacum officianalis* | 11 | 0.98 | 2.94 |  | <0.01 |  | -0.01 |  |  |  |  |  |  |
|  |  | Conifer | 17 | 0.94 | 8.07 |  | 0.01 |  | -0.02 |  |  |  |  |  |  |
|  |  | Perennial graminoids | 80 | 0.75 | 1.84 |  | <0.01 |  |  |  |  | 0.01 |  | -0.01 |  |
|  |  | Deciduous shrubs | 21 | 0.78 | 5.60 |  | <0.01 |  | -0.02 |  |  |  |  |  |  |
|  |  | Perennial forbs | 354 | 0.74 | 1.75 |  | <0.01 |  |  |  |  | 0.02 |  | -0.02 |  |
|  |  | Evergreen shrubs | 22 | 0.94 | -3.45 |  | -0.01 |  | 0.02 |  |  |  |  |  |  |
|  |  | Subshrub | 18 | 0.92 | 2.99 |  | <0.01 |  | <0.01 |  |  |  |  |  |  |

Table A3. Number of samples assayed for sequential fiber, crude protein, tannin precipitation (BSA), and gross energy (GE) from Asotin Creek, Washington, USA, and Jackson and Whiskey Mountain, Wyoming, USA, 2021–2022. Dashes indicate that no samples were submitted for that analysis.

|  | Asotin Creek | | | |  | Jackson | | | |  | Whiskey Mountain | | | |
| --- | --- | --- | --- | --- | --- | --- | --- | --- | --- | --- | --- | --- | --- | --- |
| Year | Sequential fiber | Crude protein | BSA | GE |  | Sequential fiber | Crude protein | BSA | GE |  | Sequential fiber | Crude protein | BSA | GE |
| 2021 | 502 | 431 | 63 | - |  | - | - | - | - |  | 237 | 223 | 50 | - |
| 2022 | 927 | 396 | 37 | - |  | 589 | 548 | - | 210 |  | - | - | - | - |
| Total | 1429 | 827 | 0 | 210 |  | 237 | 223 | 50 | 0 |  | 237 | 223 | 50 | 0 |

Table A4. Description of candidate covariates considered in generalized additive models of suitable forage biomass (i.e., the foodscape) available to bighorn sheep in Asotin Creek, Washington, USA, and Jackson and Whiskey Mountain, Wyoming, USA.

| Covariate | Description | Spatial resolution | Temporal resolution | Source |
| --- | --- | --- | --- | --- |
| Soil Moisture | Soil moisture in mm derived from soil water balance model | 4 km | Annual | Abatzoglou et al. [24] |
| Max Temperature | Daily maximum air temperature in degrees Celsius | 1 km | Daily | Daymet |
| Solar Radiation | Shortwave radiation in watts/m^2^ | 1 km | Daily | Daymet |
| Cumulative Precip | Cumulative daily precipitation since March 1 | 1 km | Daily | Daymet |
| Mean Temperature | Mean daily temperature since March 1 | 1 km | Daily | Daymet |
| Normalized Difference Vegetation Index [NDVI] | Vegetation greenness | 250 m | 8 days | NASA MODIS Terra |
| Enhanced Vegetation Index [EVI] | Vegetation greenness with atmospheric corrections | 250 m | 8 days | NASA MODIS Terra |
| Slope | Steepness in degrees | 30 m | - | DEM |
| Cosine Aspect | Cosine transformation of aspect [northness] | 30 m | - | DEM |
| Sine Aspect | Sine transformation of aspect [eastness] | 30 m | - | DEM |
| Elevation [DEM] | Elevation in meters | 30 m | - | USDA Landfire |
| Compound Topographic Index [CTI] | Potential wetness | 30 m | - | DEM |
| Heat Load Index [HLI] | Heat load that accounts for aspect and steepness of slope | 30 m | - | McCune et al. [25] |
| Topographic Position Index [TPI] | Landforms from regional scale-relief. Values > 0 represent hills, whereas values < 0 represent valleys | 30 m | - | DEM |
| Terrain Ruggedness Index [TRI] | Mean change in elevation between grid cell and neighbors | 30 m | - | Riley et al. [26] |
| Potential Vegetation Type [PVT] | Vegetation community | 30 m | - | USDA Landfire |
|  |  |  |  |  |
|  |  |  |  |  |

Table A5. Generalized additive models (GAMs) used to predict spatiotemporal variation in the foodscape (i.e., suitable forage biomass) available to bighorn sheep in Asotin Creek, Washington, USA, and Jackson and Whiskey Mountain, Wyoming, USA.

| Study area | Top model | Adjusted *R^2^* | Deviance explained |
| --- | --- | --- | --- |
| Asotin Creek | Usable biomass ~ s(MeanTemp^3^)^a^ HLI^b^ + CumPPT^c^ + NDVI^d^ + PVT^e^ + Ownership^f^ + FireCategory^g^ + Elevation^3^ + Slope | 0.66 | 71.40% |
| Jackson | Usable biomass ~ s(Easting)^a^ + HLI^b^ + CumPPT^3,c^ CTI^3,h^ + TPI^i^ + ln(MaxTemp) + Slope + Canopy | 0.74 | 81.10% |
| Whiskey Mountain | Usable biomass ~ s(MaxTemp)^a^ + NDVI^d^ + PVT^e^ ln(SolarRad)^j^ | 0.78 | 84.80% |
| ^a^ s() = smoothing term; ^b^ HLI = heat load index; ^c^ CumPPT = cumulative precipitation; ^d^ NDVI = normalized difference vegetation index; ^e^ PVT = Potential vegetation type; ^f^ Ownership = land ownership (Public or Private); ^g^ FireCategory = Pre, During or Post Lick Creek Fire; ^h^ CTI = compound topographic index; ^i^ TPI = topographic position index; ^j^ SolarRad = solar radiation | | | |

Table A6. Regression coefficients and associated *SE*s and *P*-values for covariates included in the top generalized additive models (GAMs) for predicting spatiotemporal variation in the foodscape (i.e., suitable forage biomass) in Asotin Creek, Washington, USA, and Jackson and Whiskey Mountain, Wyoming, USA, 2019–2022. Only *P*-values are shown for smoothing terms, which are denoted with an ‘s’. Potential vegetation type (PVT) and Fire Category were categorical variables, and coefficients represent contrasts between each listed PVT and the reference PVT (Conifer) or between Pre- and Post-Fire and the reference fire category (during fire), respectively. Other covariates are described in Table A4.

| Study area | Parameter | Coefficient | *SE* | *P* |
| --- | --- | --- | --- | --- |
| Asotin Creek | PVT-Grassland | -0.64 | 1.05 | 0.55 |
|  | PVT-Riparian | 0.38 | 1.68 | 0.82 |
|  | PVT-Shrubland | 0.91 | 1.27 | 0.47 |
|  | Ownership-Public | 1.33 | 0.66 | 0.05 |
|  | FireCategory-Post | -5.67 | 3.52 | 0.11 |
|  | FireCategory-Pre | -3.79 | 2.98 | 0.21 |
|  | CumPPT | 0.01 | 0.01 | 0.07 |
|  | HLI | -3.95 | 1.21 | 0.02 |
|  | NDVI | -3.95 | 2.37 | 0.10 |
|  | Slope | -0.04 | 0.02 | 0.07 |
|  | Elevation^3^ | 0.00 | 0.00 | <0.01 |
|  | s(MeanTemp^3^) | - | - | <0.01 |
|  |  |  |  |  |
| Jackson | HLI | 454.30 | 173.50 | 0.01 |
|  | CTI^3^ | 0.32 | 0.08 | <0.01 |
|  | Slope | 2.08 | 2.47 | 0.41 |
|  | CumPPT^3^ | 0.00 | 0.00 | 0.04 |
|  | TPI | 48.11 | 15.04 | <0.01 |
|  | Canopy | -3.66 | 1.02 | <0.01 |
|  | ln(MaxTemp) | 242.30 | 152.70 | 0.12 |
|  | s(Easting) | - | - | <0.01 |
|  |  |  |  |  |
| Whiskey Mountain | ln(SolarRad) | 169.32 | 46.91 | 0.01 |
|  | PVT-Grassland | -133.45 | 38.20 | <0.01 |
|  | PVT-Shrubland | -111.63 | 39.89 | 0.01 |
|  | PVT-Sparse | 9.84 | 20.45 | 0.63 |
|  | NDVI | 143.55 | 50.79 | 0.01 |
|  | s(MaxTemp) | - | - | <0.01 |
