# Supplementary material for: Should I stay or should I go? Causes and consequences of intraspecific variation in site fidelity
Source: Mov Ecol. 2025 Nov 6;13:80. doi: 10.1186/s40462-025-00606-w (PMC12590834; doi:10.1186/s40462-025-00606-w)
Supplement: Supplementary file 2 — Supplementary Material 2 [file 40462_2025_606_MOESM2_ESM.docx]

**Additional file 2: Appendix 2.** Simulation results illustrating the effect of window size (days) on the mean and coefficient of variation (CV) of inter-year distance for bighorn sheep at Asotin Creek, Washington, USA and Jackson and Whiskey Mountain, Wyoming, USA.


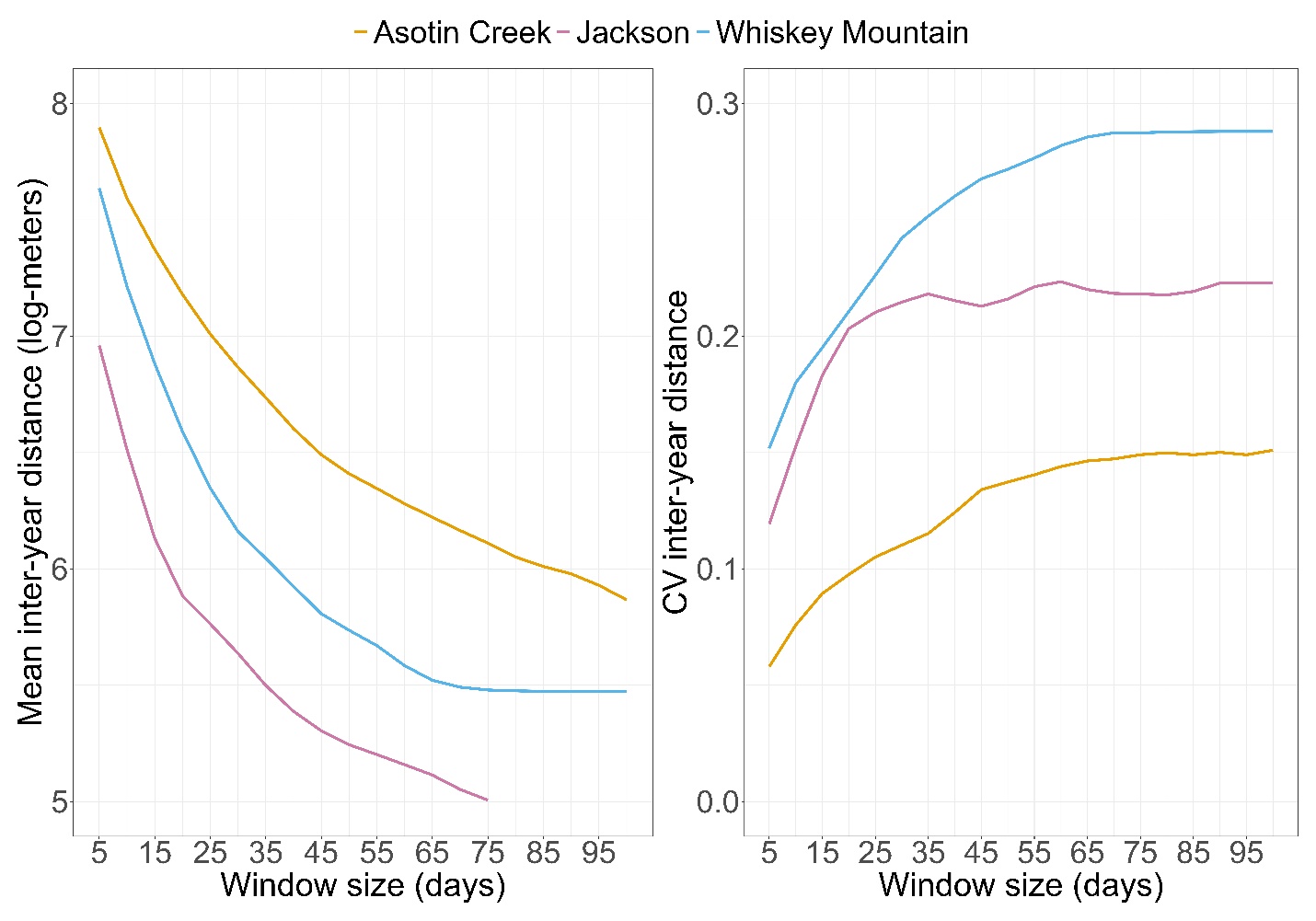


Fig. A1. Effect of window size (days) on the mean and coefficient of variation (CV) of inter-year distance for bighorn sheep at Asotin Creek, Washington, USA and Jackson and Whiskey Mountain, Wyoming, USA. We visually inspected each plot to identify the asymptote in the relationship between window size and mean or CV. We then selected the window size at which both relationships reach their respective asymptotes. We repeated this process for each study area and used the selected window size for calculations of inter-year distance [1].

**REFERENCES**

1. Morrison TA, Merkle JA, Hopcraft JGC, Aikens EO, Beck JL, Boone RB, Courtemanch AB, Dwinnell SP, Fairbanks WS, Griffith B, Middleton AD, Monteith KL, Oates B, Riotte-Lambert L, Sawyer H, Smith KT, Stabach JA, Taylor KL, Kauffman MJ. Drivers of site fidelity in ungulates. J Anim. Ecol. 2021;90:955–66.
