# Supplementary material for: Should I stay or should I go? Causes and consequences of intraspecific variation in site fidelity
Source: Mov Ecol. 2025 Nov 6;13:80. doi: 10.1186/s40462-025-00606-w (PMC12590834; doi:10.1186/s40462-025-00606-w)

**Additional file 3: Appendix 3.** Forage quality (suitable biomass) and predictability (temporal and spatial constancy) across each study area and temporal scale.


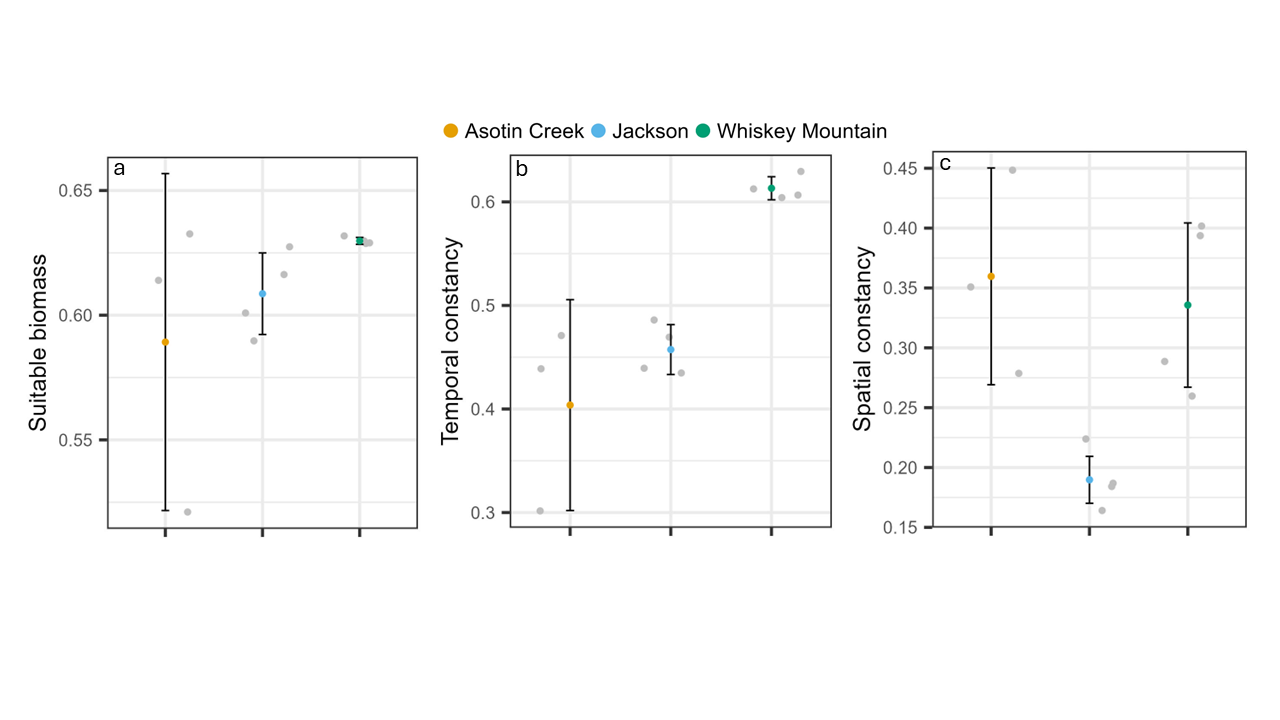


Fig. A1. Mean (colored dots), 95% confidence intervals (black bars), and raw data (grey dots) for (a) suitable biomass (i.e., home range quality), (b) temporal constancy, and (c) spatial constancy within annual population-level summer home ranges of female bighorn sheep at Asotin Creek, Washington, USA and Jackson and Whiskey Mountain, Wyoming, USA.


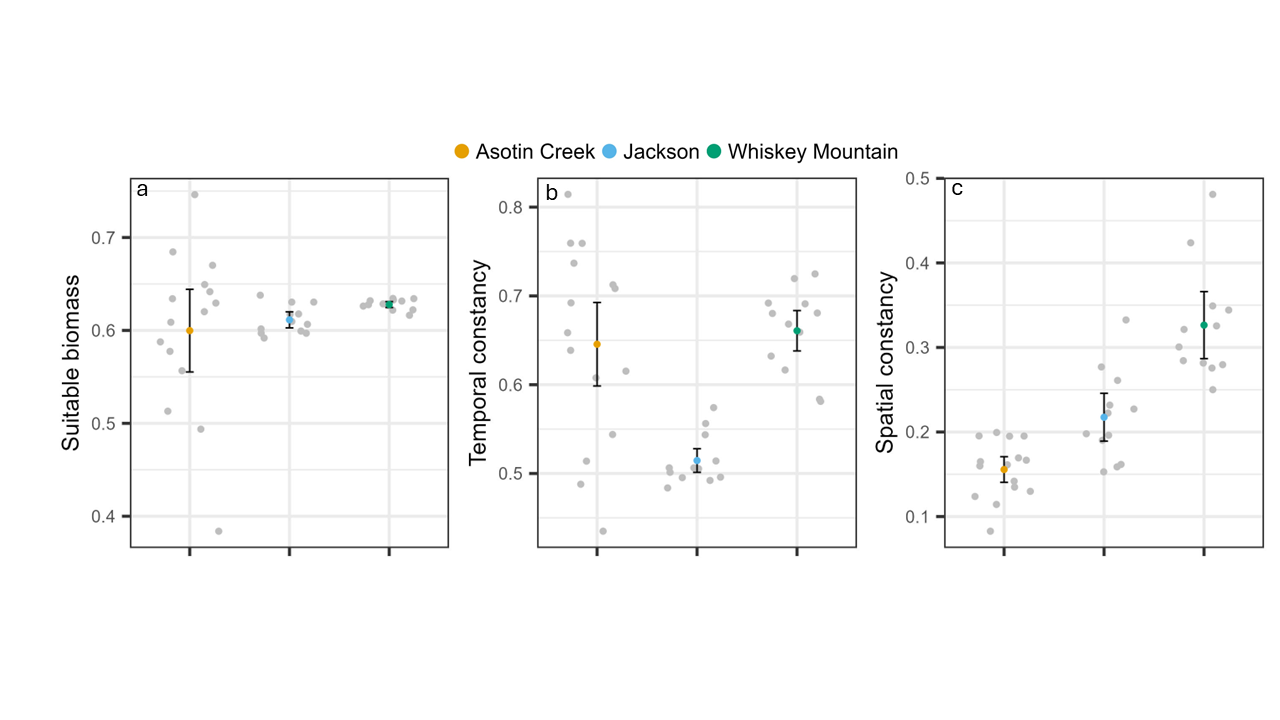


Fig. A2. Mean (colored dots), 95% confidence intervals (black bars), and raw data (grey dots) for (a) suitable biomass (i.e., home range quality), (b) temporal constancy, and (c) spatial constancy within monthly population-level home ranges of female bighorn sheep at Asotin Creek, Washington, USA and Jackson and Whiskey Mountain, Wyoming, USA.


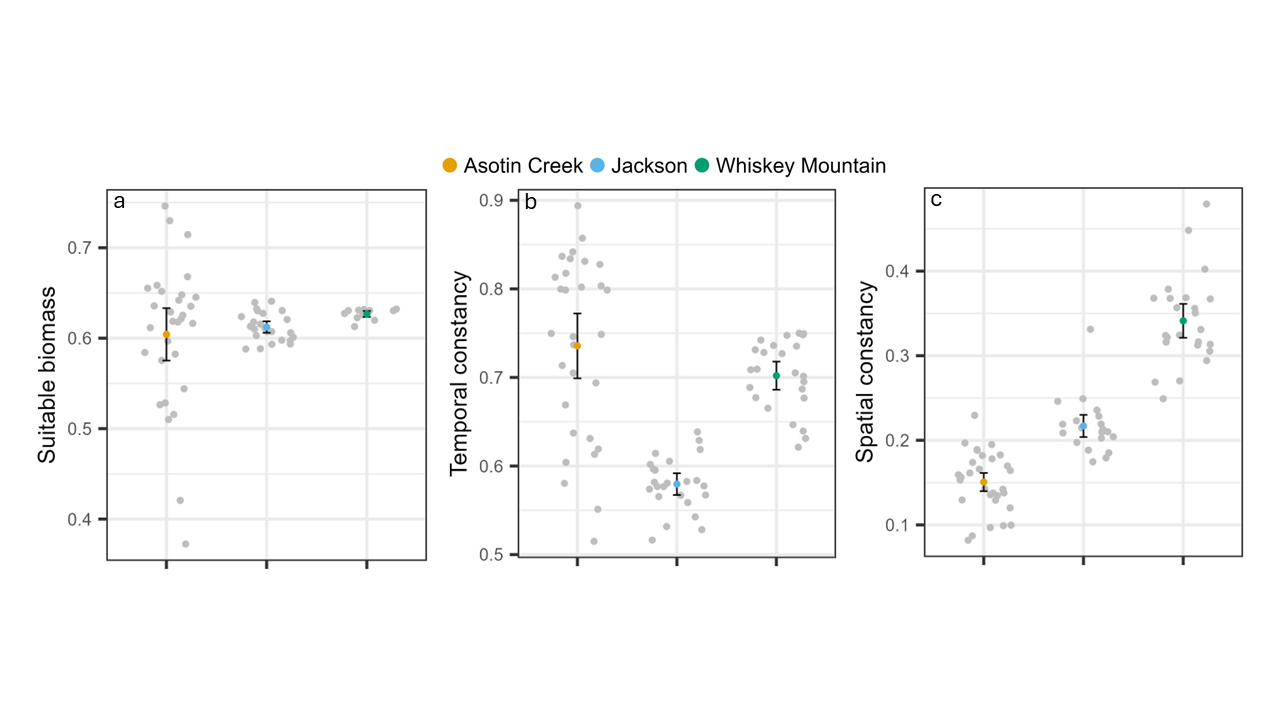


Fig. A3. Mean (colored dots), 95% confidence intervals (black bars), and raw data (grey dots) for (a) suitable biomass (i.e., home range quality), (b) temporal constancy, and (c) spatial constancy within biweekly population-level home ranges of female bighorn sheep at Asotin Creek, Washington, USA and Jackson and Whiskey Mountain, Wyoming, USA.


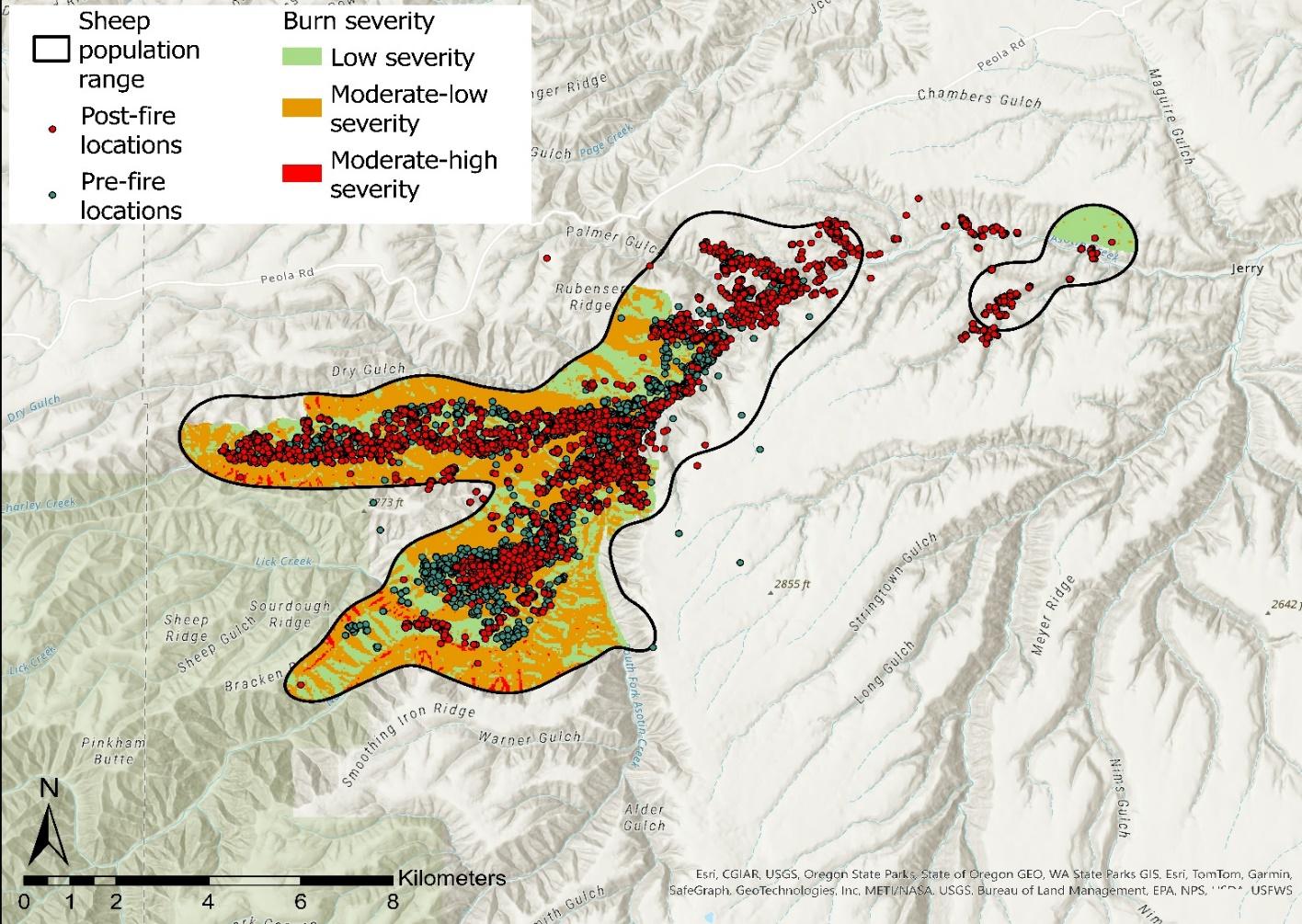


Fig. A4. Spatial variation in fire severity within the summer population range of bighorn sheep in Asotin Creek, Washington, USA after the Lick Creek Fire in July 2021. Green points show locations of GPS-collared sheep in the two weeks prior to the fire, whereas red points show locations of sheep in the two weeks after the fire.


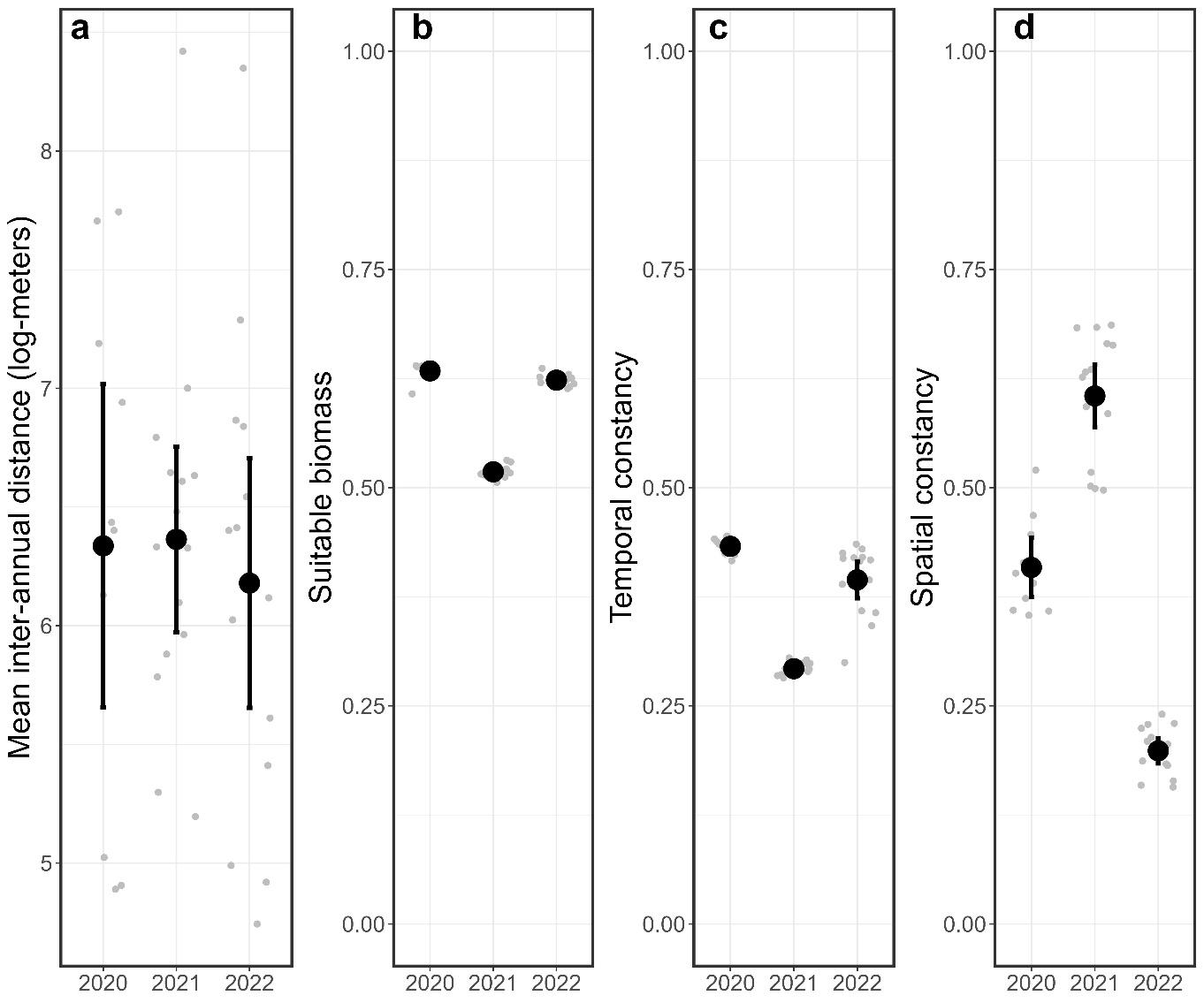


Fig. A5. Mean (black dots), 95% confidence intervals (black bars), and raw data (grey dots) for (a) inter-annual distance (i.e., site fidelity), (b) suitable biomass (i.e., home range quality), (c) temporal constancy, and (d) spatial constancy within summer home ranges of female bighorn sheep at Asotin Creek, Washington, USA, 2020–2022. Greater inter-annual distances correspond to lower levels of site fidelity across years.


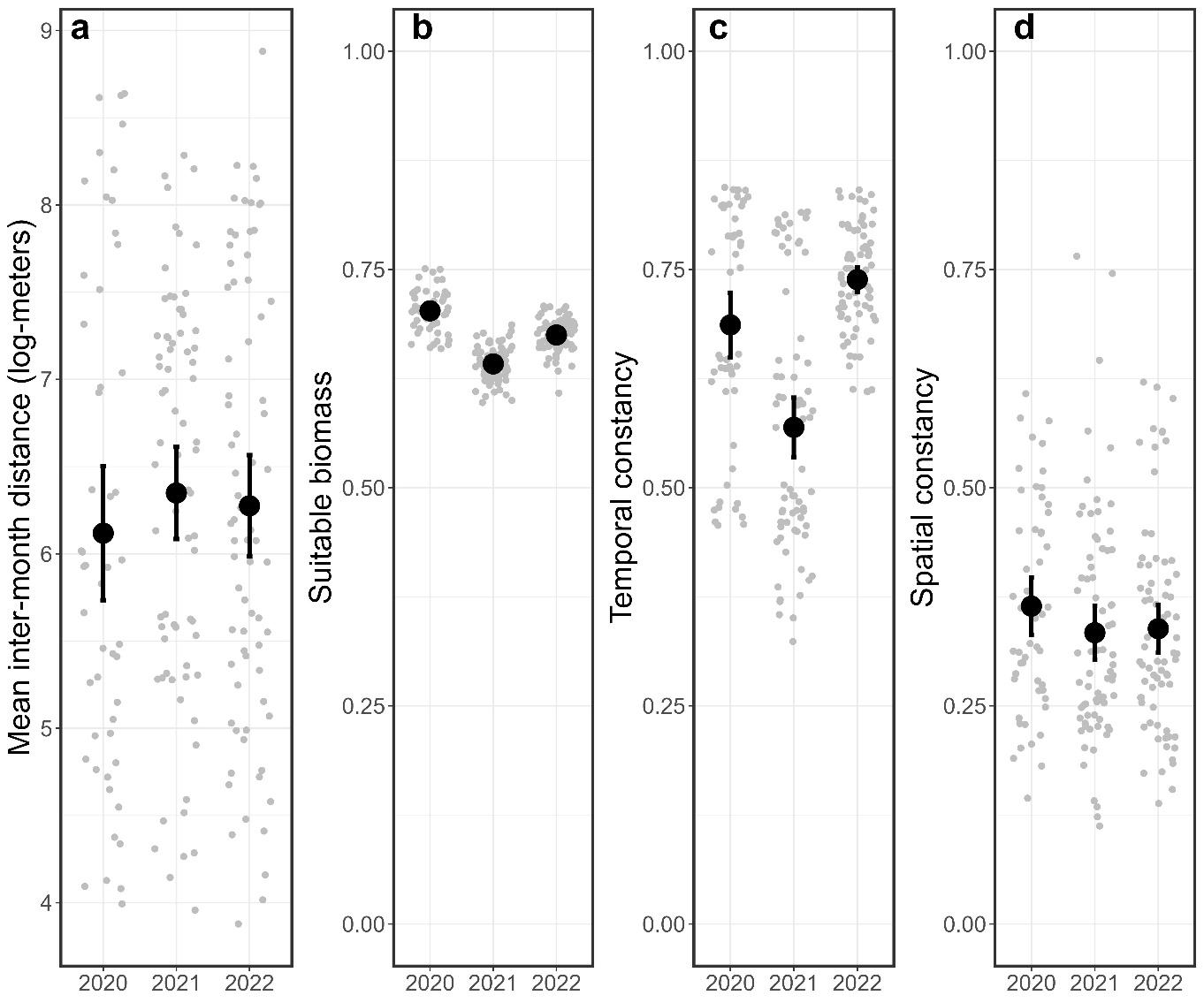


Fig. A6. Mean (black dots), 95% confidence intervals (black bars), and raw data (grey dots) for (a) inter-month distance (i.e., site fidelity), (b) suitable biomass (i.e., home range quality), (c) temporal constancy, and (d) spatial constancy within monthly home ranges of female bighorn sheep at Asotin Creek, Washington, USA, 2020–2022. Greater inter-month distances correspond to lower levels of site fidelity across months.

Fig. A7. Mean (black dots), 95% confidence intervals (black bars), and raw data (grey dots) for (a) inter-week distance (i.e., site fidelity), (b) suitable biomass (i.e., home range quality), (c) temporal constancy, and (d) spatial constancy within biweekly home ranges of female bighorn sheep at Asotin Creek, Washington, USA, 2020–2022. Greater inter-week distances correspond to lower levels of site fidelity across weeks.
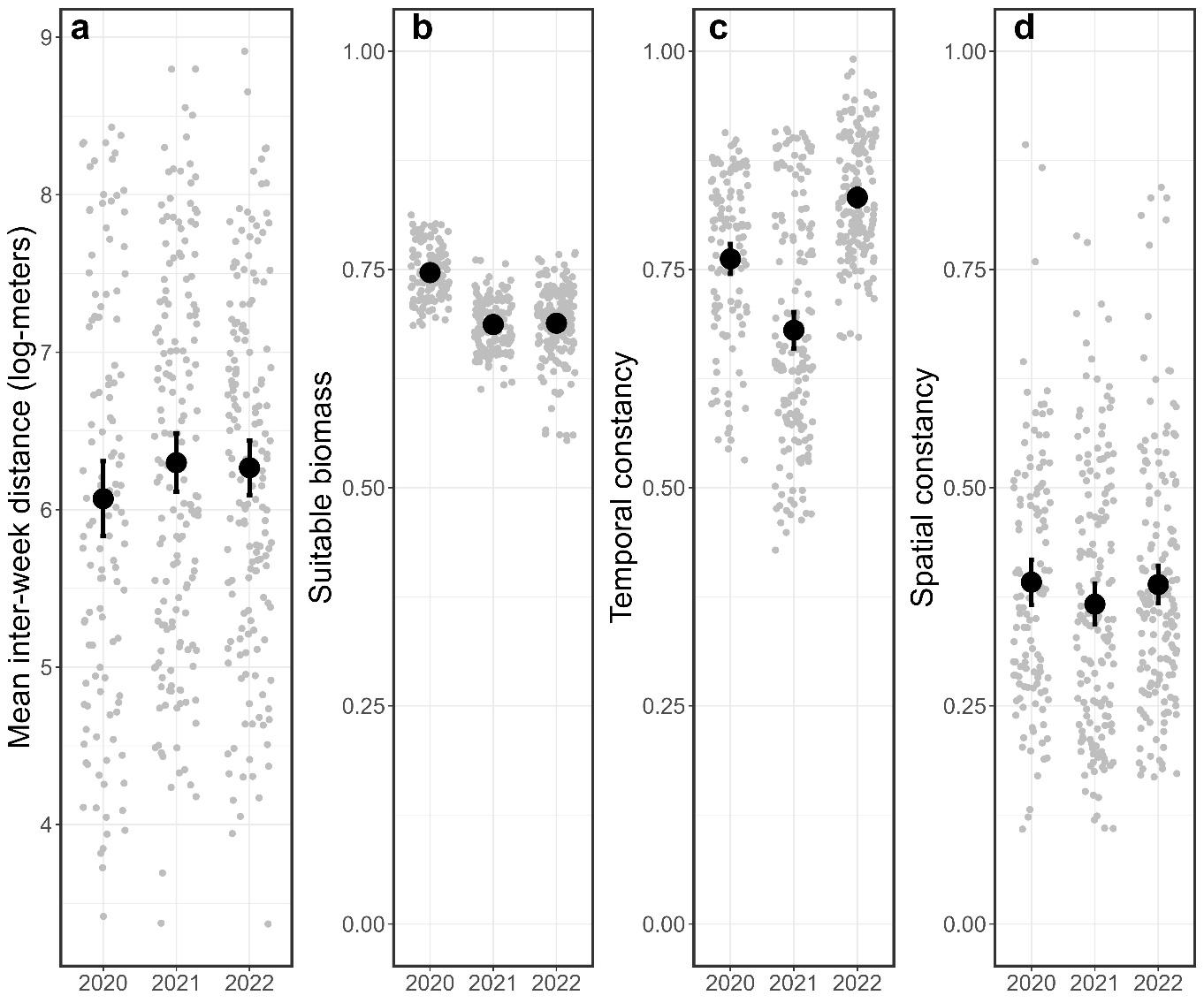

Supplement: Supplementary file 3 — Supplementary Material 3 [file 40462_2025_606_MOESM3_ESM.docx]
