# Supplementary material for: Should I stay or should I go? Causes and consequences of intraspecific variation in site fidelity
Source: Mov Ecol. 2025 Nov 6;13:80. doi: 10.1186/s40462-025-00606-w (PMC12590834; doi:10.1186/s40462-025-00606-w)
Supplement: Supplementary file 4 — Supplementary Material 4 [file 40462_2025_606_MOESM4_ESM.docx]

**Additional file 4: Appendix 4**. Model selection results for models of inter-annual, inter-month, and inter-week variation in site fidelity of bighorn sheep at Asotin Creek, Washington, USA, and Jackson and Whiskey Mountain, Wyoming, USA.

Table A1. 95% confidence sets of linear mixed-effects models quantifying the effects of environmental predictability (i.e., temporal and spatial constancy), home range quality (i.e, suitable biomass), recruitment success (WSLS), and home range size (HR) on inter-annual, inter-month, and inter-week site fidelity of female bighorn sheep at Asotin Creek, Washington, USA. The number of parameters (*k*), AIC corrected for small sample size (AIC*c*), ΔAIC*c*, Akaike weight (*w_i_*), and cumulative model weights are shown for each model. Temporal constancy (TC) and spatial constancy (SC) are included in separate models when they were correlated (*r >* 0.6).

| Scale | Candidate model | *k* | AIC*_c_* | ΔAIC*_c_* | *w_i_* | Cumulative weight |
| --- | --- | --- | --- | --- | --- | --- |
| Seasonal | TC + HR | 5 | 105.16 | 0.00 | 0.35 | 0.35 |
|  | SC + HR | 5 | 106.46 | 1.30 | 0.18 | 0.53 |
|  | Quality + HR | 5 | 106.70 | 1.54 | 0.16 | 0.69 |
|  | WSLS + HR | 5 | 107.53 | 2.38 | 0.11 | 0.80 |
|  | TC + WSLS + HR | 6 | 107.68 | 2.53 | 0.10 | 0.90 |
|  | SC + WSLS + HR | 6 | 108.81 | 3.65 | 0.06 | 0.96 |
|  |  |  |  |  |  |  |
| Monthly | Predictability + Quality + HR | 7 | 528.02 | 0.00 | 0.30 | 0.30 |
|  | Predictability + HR | 6 | 528.43 | 0.41 | 0.24 | 0.54 |
|  | Predictability + Quality + WSLS + HR | 8 | 528.51 | 0.49 | 0.23 | 0.77 |
|  | Predictability + WSLS + HR | 7 | 528.66 | 0.64 | 0.22 | 0.99 |
|  |  |  |  |  |  |  |
| Biweekly | WSLS + HR | 5 | 492.32 | 0.00 | 0.35 | 0.35 |
|  | Quality + WSLS + HR | 6 | 494.02 | 1.70 | 0.15 | 0.50 |
|  | Predictability + WSLS + HR | 7 | 494.26 | 1.94 | 0.13 | 0.63 |
|  | Predictability + HR | 6 | 494.34 | 2.02 | 0.13 | 0.76 |
|  | Quality + HR | 5 | 494.71 | 2.39 | 0.11 | 0.87 |
|  | Predictability + Quality + WSLS + HR | 8 | 495.66 | 3.34 | 0.07 | 0.94 |
|  | Predictability + Quality + HR | 7 | 495.89 | 3.57 | 0.06 | 1.00 |

Table A2. 95% confidence sets of linear mixed-effects models quantifying the effects of environmental predictability (i.e., temporal and spatial constancy), home range quality (i.e, suitable biomass), recruitment success (WSLS), and home range size (HR) on inter-annual, inter-month, and inter-week site fidelity of female bighorn sheep at Jackson, Wyoming, USA. The number of parameters (*k*), AIC corrected for small sample size (AIC*c*), ΔAIC*c*, Akaike weight (*w_i_*), and cumulative models weights are shown for each model.

| Scale | Candidate model | *k* | AIC*_c_* | ΔAIC*_c_* | *w_i_* | Cumulative weight |
| --- | --- | --- | --- | --- | --- | --- |
| Seasonal | Quality + HR | 5 | 80.17 | 0.00 | 0.61 | 0.61 |
|  | Predictability + HR | 6 | 81.89 | 1.72 | 0.26 | 0.87 |
|  | Quality + WSLS + HR | 6 | 83.89 | 3.72 | 0.09 | 0.96 |
|  |  |  |  |  |  |  |
| Monthly | Predictability + WSLS + HR | 7 | 190.90 | 0.00 | 0.36 | 0.36 |
|  | Predictability + HR | 6 | 191.96 | 1.06 | 0.21 | 0.57 |
|  | WSLS + HR | 6 | 192.70 | 1.79 | 0.15 | 0.72 |
|  | Predictability + Quality + WSLS + HR | 8 | 193.48 | 2.52 | 0.10 | 0.82 |
|  | Quality + WSLS + HR | 6 | 194.09 | 3.19 | 0.07 | 0.89 |
|  | Predictability + Quality + HR | 7 | 194.24 | 3.34 | 0.07 | 0.96 |
|  |  |  |  |  |  |  |
| Biweekly | Predictability + HR | 6 | 341.14 | 0.00 | 0.40 | 0.40 |
|  | Predictability + WSLS + HR | 7 | 341.69 | 0.55 | 0.30 | 0.70 |
|  | Predictability + Quality + HR | 7 | 342.95 | 1.81 | 0.16 | 0.86 |
|  | Predictability + Quality + WSLS + HR | 8 | 343.28 | 2.14 | 0.14 | 1.00 |

Table A3. 95% confidence sets of linear mixed-effects models quantifying the effects of environmental predictability (i.e., temporal and spatial constancy), home range quality (i.e, suitable biomass), recruitment success (WSLS), and home range size (HR) on inter-annual, inter-month, and inter-week site fidelity of female bighorn sheep at Whiskey Mountain, Wyoming, USA. The number of parameters (*k*), AIC corrected for small sample size (AIC*c*), ΔAIC*c*, Akaike weight (*w_i_*), and cumulative models weights are shown for each model.

| Scale | Candidate model | *k* | AIC*_c_* | ΔAIC*_c_* | *w_i_* | Cumulative weight |
| --- | --- | --- | --- | --- | --- | --- |
| Seasonal | Quality + HR | 5 | 54.69 | 0.00 | 0.45 | 0.45 |
|  | Quality + WSLS + HR | 6 | 56.53 | 1.84 | 0.18 | 0.63 |
|  | Predictability + HR | 6 | 56.68 | 1.99 | 0.17 | 0.79 |
|  | SC + Quality + HR | 6 | 57.47 | 2.79 | 0.11 | 0.90 |
|  | Predictability + WSLS + HR | 7 | 59.09 | 4.40 | 0.05 | 0.95 |
|  |  |  |  |  |  |  |
| Monthly | Quality + WSLS + HR | 6 | 198.54 | 0.00 | 0.46 | 0.46 |
|  | Quality + HR | 5 | 199.50 | 0.96 | 0.28 | 0.74 |
|  | Predictability + Quality + WSLS + HR | 8 | 201.95 | 3.40 | 0.08 | 0.82 |
|  | Predictability + Quality + HR | 7 | 202.16 | 3.62 | 0.08 | 0.89 |
|  | Predictability + HR | 6 | 203.00 | 4.46 | 0.05 | 0.94 |
|  |  |  |  |  |  |  |
| Biweekly | Predictability + HR | 6 | 335.54 | 0.00 | 0.34 | 0.34 |
|  | Predictability + WSLS + HR | 7 | 336.90 | 1.37 | 0.17 | 0.51 |
|  | Predictability + Quality + HR | 7 | 337.09 | 1.55 | 0.16 | 0.67 |
|  | Quality + HR | 5 | 337.83 | 2.29 | 0.11 | 0.78 |
|  | WSLS + HR | 5 | 338.01 | 2.47 | 0.10 | 0.88 |
|  | Predictability + Quality + WSLS + HR | 8 | 338.78 | 3.24 | 0.07 | 0.95 |
